# Development of Targeted Alpha Particle Therapy for Solid Tumors

**DOI:** 10.3390/molecules24234314

**Published:** 2019-11-26

**Authors:** Narges K. Tafreshi, Michael L. Doligalski, Christopher J. Tichacek, Darpan N. Pandya, Mikalai M. Budzevich, Ghassan El-Haddad, Nikhil I. Khushalani, Eduardo G. Moros, Mark L. McLaughlin, Thaddeus J. Wadas, David L. Morse

**Affiliations:** 1 Department of Cancer Physiology, H. Lee Moffitt Cancer Center & Research Institute, Tampa, FL 33612, USA; Narges.Tafreshi@moffitt.org (N.K.T.); mldoligalski@gmail.com (M.L.D.); Christopher.Tichacek@moffitt.org (C.J.T.); Eduardo.Moros@moffitt.org (E.G.M.); 2 Department of Radiation Oncology, H. Lee Moffitt Cancer Center & Research Institute, Tampa, FL 33612, USA; 3 Department of Physics, University of South Florida, Tampa, FL 33612, USA; 4 Department of Cancer Biology, Wake Forest University Health Sciences, Winston-Salem, NC 27157, USA; dapandya@wakehealth.edu (D.N.P.); twadas@wakehealth.edu (T.J.W.); 5 Small Animal Imaging Laboratory, H. Lee Moffitt Cancer Center & Research Institute, Tampa, FL 33612, USA; Mikalai.Budzevich@moffitt.org; 6 Depts. of Diagnostic Imaging and Interventional Radiology, H. Lee Moffitt Cancer Center & Research Institute, Tampa, FL 33612, USA; Ghassan.ElHaddad@moffitt.org; 7 Department of Cutaneous Oncology, H. Lee Moffitt Cancer Center & Research Institute, Tampa, FL 33612, USA; Nikhil.Khushalani@moffitt.org; 8 Department of Oncologic Sciences, University of South Florida, Tampa, FL 33612, USA; 9 Department of Pharmaceutical Sciences, West Virginia University, Health Sciences Center, Morgantown, WV & Modulation Therapeutics Inc., 64 Medical Center Drive, Morgantown, WV 26506, USA; mark.mclaughlin@hsc.wvu.edu

**Keywords:** targeted alpha-particle therapy, solid tumors, mechanism of cell death, targeting moieties, chelation, radiation dosimetry, medicinal chemistry, clinical studies

## Abstract

Targeted alpha-particle therapy (TAT) aims to selectively deliver radionuclides emitting α-particles (cytotoxic payload) to tumors by chelation to monoclonal antibodies, peptides or small molecules that recognize tumor-associated antigens or cell-surface receptors. Because of the high linear energy transfer (LET) and short range of alpha (α) particles in tissue, cancer cells can be significantly damaged while causing minimal toxicity to surrounding healthy cells. Recent clinical studies have demonstrated the remarkable efficacy of TAT in the treatment of metastatic, castration-resistant prostate cancer. In this comprehensive review, we discuss the current consensus regarding the properties of the α-particle-emitting radionuclides that are potentially relevant for use in the clinic; the TAT-mediated mechanisms responsible for cell death; the different classes of targeting moieties and radiometal chelators available for TAT development; current approaches to calculating radiation dosimetry for TATs; and lead optimization via medicinal chemistry to improve the TAT radiopharmaceutical properties. We have also summarized the use of TATs in pre-clinical and clinical studies to date.

## 1. Introduction

**Figure 1:**
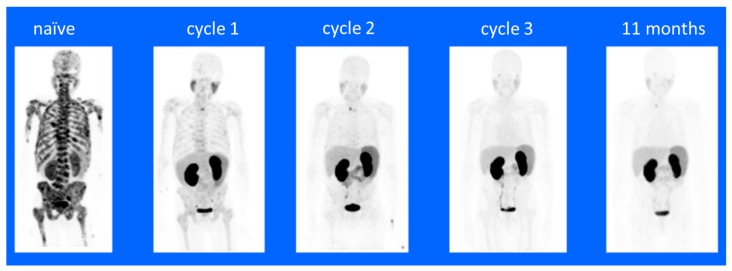
^68^Ga-PSMA-11 PET/CT images of a treatment-naïve patient with extensive bone metastasis at primary diagnosis. A complete remission was observed after three cycles of ^225^Ac-PSMA-617 with de-escalating activities of 8/7/6 MBq. The patient remained symptom-free with undetectable serum PSA and a negative ^68^Ga-PSMA-11 PET/CT at 11-month follow-up evaluation. This figure and legend were reproduced from Sathekge, et al. [6].

Over the past two decades, radioimmunotherapy (RIT) has proven to be an effective treatment for non-solid tumors (reviewed in [1,2]); e.g., radiolabeled anti-CD20 monoclonal antibodies for the treatment of lymphoma. These antibody-radionuclide conjugates have typically used beta (β)-particle emitting radionuclides; e.g., ^131^I, ^67^Cu, ^177^Lu or ^90^Y. However, due to the relatively long range of the associated β-emissions and the poor tumor penetration of antibodies, there has been concern regarding the use of RIT for treatment of solid tumors, where much of the energy is deposited in the surrounding normal tissues relative to the tumor, particularly in the case of small tumor cell foci or metastases [1]. Alpha (α)-particle-emissions have a much shorter range and greater linear energy transfer (LET) relative to β-emissions, depositing more energy into smaller volumes [3]. Hence, there has been significant interest in the development of targeted alpha-particle therapy (TAT) for the treatment of solid tumors. Recently, the FDA approved the use of ^223^RaCl_2_ (Xofigo^®^) for the palliative care of prostate bone metastases, and the efficacy of ^223^RaCl_2_ and ^225^Ac–PSMA–617 have been demonstrated in the treatment of prostate bone metastases (Figure 1) [4,5,6]. These developments have further elevated interest in the development of novel α-emission cancer treatments [7,8]. Typically, TAT for solid tumors involves attaching an α-particle-emitting radionuclide to a tumor targeting scaffold, followed by the intravenous administration and systemic targeting of tumors and metastases. The α-particle range is only a few cell diameters, ensuring that the greatest effect of tumor TAT remains within the tumor volume [3]. Herein we discuss the current progress, challenges and approaches toward the development of novel TATs for the treatment of solid tumors.

There are differences in the mechanism of tumor cell killing when comparing β-emission to α-emission therapies. β-particles scale to the size of electrons, travel over a relatively longer range (0.5–12 mm) in tissues in comparison to α-particles, have relatively lower LET and generate hydroxyl free-radicals by breaking covalent bonds of water molecules in the tissue [9]. These free radicals result in oxidative damage to the cellular DNA macromolecules, causing double-strand breaks [10]. In contrast, α-particles are heavier (size of He atom), travel over a much shorter range (40–90 µm), and thus, have hundreds fold higher LET (α = 100 keV/µm versus β = 0.2 keV/µm) [11,12,13,14]. Hence, α-emissions deposit large amounts of energy in a smaller volume relative to β-emissions and result in the direct breaking of covalent bonds; e.g., DNA double-strand breaks. In either case (α or β), the DNA damage can activate DNA damage checkpoints and double-strand break repair [15]. Where the damage is significant or if there are defects in the checkpoint or repair pathways such that repairs cannot be made, i.e., irreparable damage, programmed cell death (apoptosis) is initiated [16]. In apoptosis-deficient tumor cells, the resulting damage to the cellular machinery eventually results in necrotic cell death [16]. Hence, α-emission therapy potentially has several distinct advantages relative to β therapy. First, the shorter range of α-emissions result in lower off-target damage but will still allow for killing of adjacent tumor cells. Since tumors are known to be heterogeneous in marker expression [17], nearby cells that do not express the target can also be damaged and killed, potentially eliminating a mechanism of resistance; i.e., killing of marker expressing cells with the clonal expansion of non-marker expressing cells. Second, the greater energy deposition will result in greater DNA damage and a correspondingly greater level of cell killing. Third, α-emission-mediated DNA damage does not rely on the generation of free radicals, potentially eliminating the development of a major mechanism of resistance to radiation therapy; e.g., upregulation of superoxide dismutase, etc. [3]. 

There are many α-emitting radionuclides available for use in the development of TAT, and factor, including nuclear characteristics, availability, chemistry, specific activity, synthesis yield, chemical and biological stability of conjugates, and costs, need to be considered. Choice of targeting scaffold is also a major consideration in TAT development. Antibodies, antibody fragments, peptides and passive targeting strategies have been employed and the type of targeting moiety chosen will have a bearing on the type of radionuclide attachment used, the route of clearance and blood pharmacokinetics (PK), tissue biodistribution (BD) and radiation dosimetry. The relative merits of available radionuclides, attachment chemistries and targeting moieties are discussed herein. Radiation dosimetry (RD) is an area of key importance in development of TATs. The discrete decay chains of some α-particle-emitting radionuclides involve the generation of daughter products with individual radioactive emission properties. Hence, the biological fate of the daughter products and resulting tissue exposure to ionizing radiation is a major concern that must be evaluated and the current status of RD studies in the context of TAT development are also discussed. Strategies for lead optimization via medicinal chemistry to improve the TAT radiopharmaceutical properties and methods of current good manufacturing practice (cGMP) production are discussed. Also included are brief reviews of preclinical and clinical TAT studies conducted to date. 

## 2. Mechanism of Action/Tumor Cell Killing

Since the discovery of radioactive materials, the effect of radiation on the properties of matter has been of significant interest in the disciplines of material science, geology and astrophysics. For example, the first large-scale effect of radiation on solid material was observed by E.P. Wigner via exposure to a nuclear fission reactor, and that was thus named the Wigner effect, or “Wigner’s disease” [1]. Since then, the large spread of medical technology involving sources of ionization radiation [2] and the development of nuclear weapons has caused a spike in studies on the effect of radiation on living tissue. That work increased our understanding of the concept of radiation risk and created new fields of scientific study; for example, radiation health safety, radiation dosimetry and radiation oncology. 

Because of the high energy of α-particles and stochastic nature of ionization radiation, their effects may be observed on all levels of a biological system. Any molecule, cell, tissue or organ can display α-decay radiation damage, and such damage can be localized, or occur throughout the entire body of any multicellular organism [3]. 

The first step in producing radiation effects is the generation of a primary recoil atom and α-particles by a radioactive decay. Such events take place very rapidly in much less than 1 fs [4]. In the case of α-decay of radiopharmaceutical isotopes of interest, the average kinetic energy per recoil atom is ~100 keV, and the average kinetic energy deposited within the range of a single α-particle is 5 MeV. It is clear that the relatively high levels of energy deposited by the combination of the fast-moving heavy ions and high energy α-particles can cause large amounts of damage to solid matter. There are many methods to estimate the effect of radiation in solid materials; e.g., the stopping theory based on coulombic interactions, molecular dynamics and transport theory. However, unlike solid materials, biological tissues do not consist of solid crystalline structures, and this significantly increases the complexity of estimating the effects of α-decay (as well as other ionization radiation), making the application of the aforementioned methods impossible or extremely difficult numerically. To overcome such limitations, a semi-quantitative approach has been applied. First, the biological effects observed in irradiated subjects were separated into one of two categories [3,6]: deterministic effects, which have a practical threshold absorption dose below which effects are negligible or not evident; and stochastic effects, where the relationship between dosage and severity of effect is either less evident or absent.

**Figure 2:**
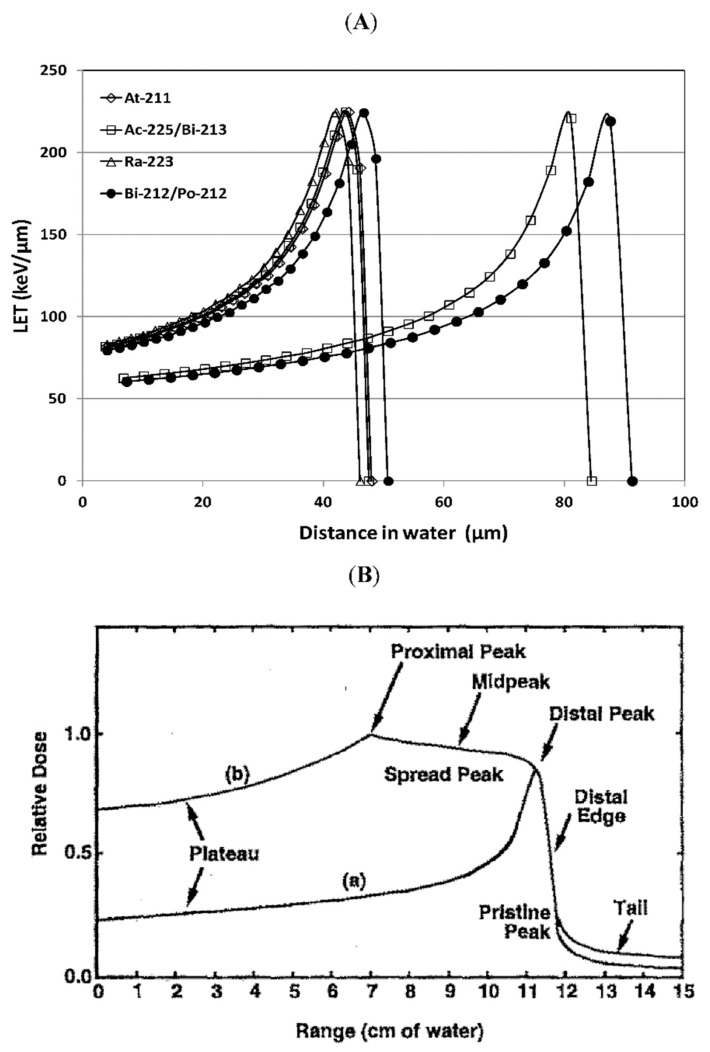
(**A**) Linear energy transfer (LET) versus distance in water traveled by typical α-particles emitted by radionuclides in development for α-particle radioimmunotherapy: ^225^Ac (5.829 MeV)/^213^Bi (8.375 MeV), ^211^At (5.867 MeV), ^212^Bi (6.08 MeV)/^212^Po (8.78 MeV), ^223^Ra (5.716 MeV). The range of the α-particle and the position of the Bragg peaks are correlated with the initial energy of the α-particles. LET of α-particles in water was calculated using stopping-power and range tables (continuous slowing down approximation range) for electrons, protons and helium ions from the National Institute of Standards and Technology (NIST). (**B**) The deposition of heavy ion energy as a function of penetrating depth of (a) a pristine beam and (b) a modulated beam with widened stopping region (spread out Bragg peaks). This figure and legend were reproduced from [19].

Maintaining the integrity of many different types of macromolecular structures is important to cell viability and all cellular organic molecules are subject to damage by ionizing radiation. However, the genomic DNA molecules are considered to be the most critical targets for the biological effects of ionizing radiation because intact DNA is required for cellular replication and damaged but repaired DNA can result in the fixation of genetic mutations that can affect normal cellular function and viability [18]. Ionizing radiation interacts with DNA either by directly transferring energy to the biological material or indirectly by creating reactive free radicals from the radiolysis of water. These interactions result in damage to the DNA’s structure via broken covalent bonds. Linear energy transfer (LET) is an approach to describe the spatial distribution of ionization and excitation produced by direct or indirect effects of different types of radiation along a linear path [15]. Alpha (α) particles have high LET radiation because they create dense ionizations and excitations in matter due to coulombic interactions with atoms. Being a heavy charged particle, an α-particle will continuously slow down along its track with minimal deflection. Through the process of slowing down, the interaction cross-section towards the end of travel increases, resulting in increased LET, which is known as the Bragg Peak (Figure 2) [19,20]. 

Relative biological effectiveness (RBE) is the ratio of the dose of a reference radiation and the dose of a test radiation that produces the same biological effect. Some of the most common biological effect measurements are necrotic and apoptotic (programmed) cell death, DNA damage, chromosomal aberrations and genetic mutations. The RBE of α-particles can range from 3.5 to 4 for cell killing or 6 to 12 for mutation, and be up to 10 for cell transformation [15]. As a comparison, the RBE for low LET electrons and photons is 1.

**Figure 3:**
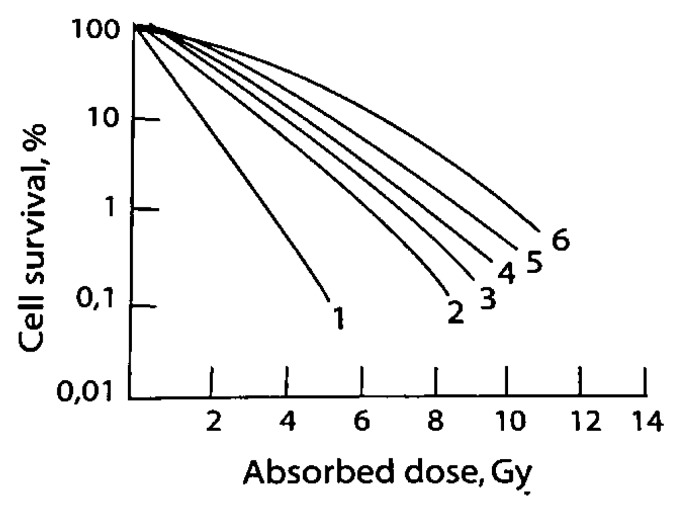
Survival of a human kidney T-cell culture irradiated with ionizing particles of different kinds: (1) particles with E = 2.5 MeV, LET = 165 keV/m; (2) particles with E = 27 MeV, LET = 25 keV/m; (3) deuterons with E = 3.0 MeV, LET = 20 keV/m; (4) X-rays with E = 20 keV and LET = 6 keV/m; (5) X-rays with E = 250 keV and LET = 2.5 keV/m; and (6) particles with E = 2.2 MeV, LET = 0.3 keV/m. This figure and legend were reproduced from [21].

An important biological endpoint is cell killing. Damage to cells can be classified as either sub-lethal events or lethal events. Sub-lethal events are due to the accumulation of damage that has the potential to be repaired, typically as a result of exposure to lower doses, and lethal events typically result from irreparable damage due to exposure to higher doses [3]. The ability to repair these events is seen as a shoulder on the cell survival curve and is characteristic of low LET radiation (Figure 3) [21]. A single event of high LET radiation can be lethal. The cell survival curve for a lethal event does not have a shoulder, indicating the inability to repair [22].

The DNA double-strand break is the most biologically significant type of damage, which occurs as a result of two single strand breaks in close proximity or a rupture of the double-strand at the site of interaction [23]. Cell survival is highly dependent on the spatial distribution of double-strand breaks [15]. Given the same dose, high LET radiation can create up to four times more double-strand breaks of low LET radiation. Additionally, the formation of high LET double-strand breaks are more complex compared to low LET in that they are less randomly distributed and form clustered DNA damage to multiple base-pairs [19]. It has been widely accepted that high oxygen levels play a large role in a cell’s sensitivity to ionizing radiation, and hence, tumor hypoxia is an established factor in resistance to radiation therapy [24]. This is due to enhancement of free radical production by the presence of oxygen. Free radical production occurs as a result of indirect action, or low LET interactions. Since α-particles interact directly with the DNA, the level of oxygen becomes irrelevant; hence, eliminating a major mechanism of resistance to therapy [3].

After exposure to radiation that results in DNA damage, the cell cycle can be stopped at cell-cycle checkpoints which allow the cell to repair the damage via multiple repair mechanisms in order to preserve genomic integrity [23]. In the case of irreparable damage, the cell will eventually undergo cell death by apoptosis or necrosis. The two main repair mechanisms of double-strand breaks are homologous recombination and non-homologous end joining. Homologous recombination occurs in the late S and G2 phases of DNA synthesis where an intact DNA template is available, resulting in more efficient and higher-fidelity repair. Non-homologous end joining occurs throughout the cell cycle but is the only means of repair in G1 and early S phases. In this error prone repair method, DNA ends are rejoined with no sister templates [22]. In this case, chromosomal aberrations can occur as a result of recombining incorrect DNA ends; i.e., combining a loose end to some other molecule, and the truncation of ends. If incorrect repair occurs prior to DNA replication, these errors can be replicated in daughter DNA which can lead to mitotic cell death or can lead to the generation of genomic mutations without cell death [22]. There is also the situation where double-strand breaks are not repaired and the dividing cell enters mitosis, leading to mitotic catastrophe and eventual cell death. A higher proportion of double-strand breaks remain un-rejoined after exposure to high LET radiation [15]. When it comes to damage from high LET α-particles in close proximity to the cells being irradiated, the main radiobiological effect is complex and irreparable DNA damage resulting in cell death by either apoptosis or necrosis.

In the last few years, successful attempts have been made to explain the bystander effect [24,25]. The bystander effect is defined as a group of effects that are observed in cells that have not been directly irradiated following the irradiation of other nearby cells. Two mechanisms were proposed. One is the transfer of genomic instability through p53-mediated pathways, and the other suggests that irradiated cells secrete cytokines or other factors that transit to other cells that are not irradiated and signal for increased levels of intracellular reactive oxygen species [26]. One of the defined sub-classes of the bystander effect is the “abscopal effect,” in which radiation treatment of a tumor propagates to tumors outside the irradiated volume [26]. A more recent publication demonstrated an effect that might explain the abscopal effect [27]. It was demonstrated that α-particle treatment of prostate cancer cells generated an adaptive antitumoral immune response, as has been previously reported for other forms of radiation. Combinations of bystander effects and the abscopal (likely immune) response in vivo are potential mechanisms of the efficacy for tumors that are not venerable to the targeted α-emitter radiotherapy in a patient with heterogeneous target expression.

## 3. Alpha-Particle Emitting Radionuclides

Using radiation as a method of cancer therapy requires delivering the maximum dose to the tumor while minimizing the dose to healthy tissues. Targeted radionuclide therapy is advantageous in that it seeks molecular and functional targets within patient tumor sites [28]. Beta (β)-emitting radionuclides (^90^Y, ^131^I, ^177^Lu and ^186^Re) are used for cancer-targeted therapy but have problems with cross-fire irradiation of normal tissues due to their relatively long range in tissue, which is 0.5–12 mm. In contrast, α-particles deposit higher energy over a much shorter range (40–90 µm), potentially causing higher cytotoxicity to tumor cells while delivering a lower dose to normal, adjacent tissues [29]. Alpha (α)-emission is the process by which an unstable nucleus ejects a highly-energetic, heavy, charged particle consisting of two protons and two neutrons. Alpha (α)-particles have a higher LET (100 keV/µm) compared to β-particles (0.2 keV/µm) which results in a dense ionization track in matter. The short range of α-emission provides specificity to the target cell population with a minimal effect on surrounding normal cells, and the high LET leads to a high frequency of irreparable DNA double-strand breaks [18]. This limits cytotoxic effects to within a small distance from the location of decay. It has been estimated that only one cell traversal by an α-particle track is necessary to kill a cell while, thousands of β-particle traversals are required for the same effect [30]. Because of the long range of β-particles and the need of a high number of hits for cell killing, a large portion of the dose deposited is outside of the intended target. In addition to treatment of solid tumors, the use of α-particles for targeted treatment of circulating disease could be an improvement due to the potentially reduced damage to normal tissues. Use of TAT has been considered for killing isolated cancer cells in transit in the vascular and lymphatic systems, in regressing tumors by disruption of tumor capillary networks and in treatment of micrometastatic foci [31]. In particular, TAT may be ideal for treatment of solid metastases, as the short range will primarily kill tumor cells, with little deleterious impact on surrounding normal tissue. Additionally, heterogeneity of target expression has been observed within a given tumor or metastasis [32] and this is thought to be a mechanism of developing resistance to targeted therapies; non-target expressing cells survive treatment and clonally expand into a resistant population. In this case, α or β-emissions from a targeted cell will serve to kill surrounding untargeted cells within the effective range, potentially reducing the development of resistance. 

A number of factors need to be considered when choosing an α-emitting radionuclide for therapy. These include proper nuclear characteristics, ease of radiochemical incorporation, specific activity, synthesis yields, chemical and biological stability, availability and cost [29]. 

The physical half-life of the radionuclide should be long enough to allow for radiosynthesis preparation and be compatible with the pharmacokinetics of tumor localization [33]. The decay pathway of the α-emitter should be carefully analyzed. Due to the conservation of energy and linear momentum, a daughter nuclide which subsequently decays by α-emission could detach from the radioimmunoconjugate; see for example [34]. These free products could travel away and deposit doses to healthy tissues. A decay chain that is long and complicated, having many different decay types, could also present an issue dosimetrically, especially if the daughter products are metabolized differently than the parent. A possible way to overcome this issue is to use ^225^Ac as an in vivo generator in which the delivery system is designed to be internalized into the target cell where the toxic daughter elements may detach from the targeting vector but remain trapped in the cell [35]. 

Another important nuclear characteristic is having a large number of α-emissions per decay. The radiotoxicity associated with having multiple emissions could be high enough to kill a tumor cell in a single decay. An accompanying gamma (γ) photon emission with energy suitable for in vivo imaging is also beneficial for assisting with pharmacokinetic and dosimetric evaluations [33].

Another important element for radionuclide selection is availability. Alpha (α)-emitters are produced either by cyclotron bombardment or by reactor irradiations, are incorporated into a generator and are eluted from a parental source. Obtaining radionuclides in pure form with high specific activity and large quantities is essential for adequate therapeutic evaluation. High specific activity is important to avoid receptor saturation by the unlabeled targeting agent [36]. If membrane antigenic density is low, insufficient binding to tumor cells will occur [36]. Transportation of these radionuclides safely and economically is also a key issue in selection.

While there are over 100 α-emitting radionuclides, only several have been investigated in preclinical and clinical studies. This is mostly due to radionuclides lacking nuclear properties, the absence of viable chemistry, complicated decay chains and production and economic issues [37]. Therefore, radionuclides meeting the criteria for therapeutic use have been limited to ^225^Ac, ^211^At, ^212^Bi, ^213^Bi, ^212^Pb, ^223^Ra, ^224^Ra, ^149^Tb and ^227^Th. The physical characteristics of these isotopes can be seen in Table 1.

The first α-emitter to be used in human clinical trials for therapy was ^213^Bi in 1997, when it was labeled to the anti-leukemia antibody HuM195 [38]. ^213^Bi is available through generator based ^225^Ac and decays with a 45.6 min half-life by emission of one α (8.37 MeV) and two β-particles. The generator is produced at Oak Ridge National Laboratory in the US and at the Institute for Transuranium Elements in Karlsruhe Europe. In the decay of ^213^Bi, there is an emission of a 440 keV isomeric γ, which is beneficial for imaging studies.

^211^At decays with a half-life of 7.2 h and emits two α-particles through a split decay pathway with energies of 5.87 and 7.45 MeV. One path is to ^207^Bi by α-emission followed by electron capture to ^207^Pb and the other is by electron capture to ^211^Po followed by α-emission to ^207^Pb. An advantage of this decay path is that ^211^Po emits 77–92 keV characteristic X-rays which can be used for imaging [37]. The main disadvantages are availability and purity. Conventionally, the production of ^211^At requires α-particle cyclotrons, which are only available in a few places worldwide, to produce the ^209^Bi(α,2n)^211^At reaction with minimal ^210^At contamination [39]. Astatine has significant metallic characteristics that lead to complications in standard antibody labeling and results in a rapid release of free ^211^At [37,40,41]. To resolve this problem, approaches have been developed by several research groups based on small linker molecules that create an aryl carbon–astatine bond involving an astatodemetallation reaction using tin, silicon or mercury precursors [42]. Other alternative methods for astatination, e.g., boron-astatine, rhodium-astatine or nanoparticle encapsulation, are also being pursued [43].

^225^Ac is a radiometal with a half-life of 10.1 days and produces six radionuclide daughters in the decay path to stable ^209^Bi. For each decay event of ^225^Ac, there are successively, four α and two β emissions with high energy (α 8.38 MeV, β 1.42 MeV). In the decay of ^225^Ac and its daughters there are several isomeric γ emissions with energy suitable for imaging studies. The relatively long half-life allows for a centralized production site that can ship ^225^Ac to users [44]. The main method for generating ^225^Ac for clinical studies is through the decay of ^229^Th which originates from ^233^U. In the world there are three main sources of ^229^Th: Oak Ridge National Laboratory (USA), The Institute of Physics and Power Engineering (Russia) and The Institute for Transuranium Elements (Germany). The quantities produced are not enough for a global application of ^225^Ac. To keep up with the increasing demand for ^225^Ac for clinical applications, it has been found that large scale quantities can be produced through high-energy proton irradiation of ^232^Th [45,46]. To address the shortage, the US Department of Energy formed a Tri-lab collaboration of Los Alamos (LANL), Brookhaven (BNL) and Oak Ridge (ORNL) National Laboratories with the goal of developing an alternative route for production of Ac [47]. Another limitation for this radionuclide has been with the radiochemical stability of the attachment to immunoconjugate. McDevitt et al. [34], state that the instability of these attachments is due to the high classical recoil energy (100–200 keV) of the daughter product which breaks the molecular bonds of the chelator. Significant advances have been made in developing chelators that form thermodynamically stable and kinetically inert complexes with ^225^Ac. Khabibullin et al., recently calculated the chelation stability of ^225^Ac and daughters in the 1,4,7,10-tetraazacyclododecane-1,4,7,10-tetraacetic acid (DOTA) chelator [48]. 

As one potentially abundant starting material, ^212^Bi (1.01 h half-life) can be obtained from ^228^Th and decays via a branched pathway to ^208^Tl (36% α) and ^212^Po (64% β); then, both decay to stable ^208^Pb [33]. However, ^212^Bi has several disadvantages that potentially limit its use. First, its short half-life can be problematic if the production and shipping processes are lengthy. This issue can be solved by using ^224^Ra as a generator to locally produce ^212^Bi. Another complication is the high energy γ emission (2.6 MeV) which requires a considerable amount of shielding to minimize exposure. This, along with the short half-life, makes shipping difficult, resulting in an availability problem. 

^223^Ra is found naturally in trace amounts following the decay of ^235^U, but it is mainly produced artificially by the decay of ^227^Th (T_1/2_ = 18.7 days), which is produced from ^227^Ac (T_1/2_ = 21.77 years). Since ^227^Ac is found only in traces in uranium and thorium ores, it is mainly synthesized by ^226^Ra (T_1/2_ = 1600 years) irradiation in a nuclear reactor [49]. ^223^Ra has a half-life of 11.4 days and emits four α-particles, two β-particles and γ rays on the path to stable ^207^Pb [34]. While the emission of four α-particles is advantageous for tumor toxicity, ^219^Rn in gaseous form is also emitted, which can redistribute in the body and dose non-targeted cells. See Figure 3 for comparable energies of α-particles. 

The major challenge using ^223^Ra is finding a suitable ligand for in vivo sequestration. However, similarly to cations of the alkaline earth elements, radium has natural bone-seeking properties without the need for a carrier agent. As radium mimics calcium, when ^223^Ra dichloride (Xofigo^®^) is injected intravenously, it forms complexes with the bone mineral hydroxyapatite at areas of increased metabolic bone activity, such as bone metastases, thereby exerting a highly localized antitumoral effect [7]. In addition, the ^223^Ra daughter isotopes are also retained in the bone matrix [50]. 

Thanks to this characteristic, ^223^Ra dichloride (^223^RaCl_2_) has demonstrated improvement in overall survival for the treatment of bone metastases in castration-resistant prostate cancer and has received FDA approval for this application [34], making it the first FDA approved TAT. 

^227^Th can be produced continuously from ^227^Ac and decays with a half-life of 18.7 days to ^223^Ra [51]. The long half-life is beneficial for radiolabeling and targeting. ^227^Th also decays with accompanying γ emissions of 236 keV and 50 keV, which can be used for imaging but are low enough to avoid the need for patient shielding.

^212^Pb has a 10.6 h half-life and is produced either from the decay chain of ^228^Th or by the ^224^Ra generator [39]. Because of its long half-life and the fact that ^212^Pb is a β-emitter that decays to ^212^Bi, one approach has been to use this radionuclide as an in vivo generator to compensate for the short half-life of ^212^Bi [52]. ^212^Pb can deliver over ten times the dose per unit of administered activity compared to ^212^Bi or ^213^Bi [53]. The major issue with ^212^Pb is the electron capture and auger electron emissions which can cause significant recoil of the ^212^Bi daughter [54]. The free ^212^Pb has been shown to cause severe bone marrow toxicity [55], while the free Bi has shown to cause kidney toxicity. By co-injecting DTPA or EDTA chelating agents, rapid release of free ^212^Bi can be achieved [56].

## 4. Targeting Moieties

In the burgeoning field of targeted radiopharmaceuticals, there is no universal answer to the question: what is the best targeting molecule? Rather, the abundance of target molecule classifications allow for a more customized approach to developing TATs. Globally, the requirements of a targeting ligand include the ability to concentrate at and bind to extracellular targets and the availability of chemical functional groups amenable to the attachment of linkers and chelators. A balance of many other factors like off-target binding, biodistribution and pharmacokinetics (particularly with respect to the decaying half-life of the chosen radionuclide) are all critical factors for the selection of a proper targeting ligand. Recently, small molecules, peptides, antibodies, antibody fragments and even some passive targeting strategies have been investigated to deliver radioisotope payloads. See Table 2 and Table 3 for examples of TAT conjugates that have been studied in the pre-clinical and clinical environments. This section will explore the advantages and challenges of the aforementioned targeting ligand categories. 

### 4.1. Small Molecules

An advantage of using low molecular weight ligands as targeting moieties for α-therapy include rapid penetration into the tumor and rapid clearance of unbound conjugates from circulation; thus, reducing toxicity. Internalizing ligands are particularly beneficial for ^225^Ac’s application in order to harness the multiple α-particles emitted in its decay chain. A successful example of using small molecules in TAT is ^225^Ac-PSMA-617 for the therapy of metastatic, castration-resistant prostate cancer [57,58]. The favorable pharmacokinetics properties of the PSMA-617 small molecule include its fast tumor uptake, high internalization rate, extended tumor retention and rapid clearance of unbound ligand [58]. These features make the molecule highly desirable for labeling with an α-emitter with a half-life of several days and multiple α-emissions in its decay chain, like ^225^Ac. The first report of the significant therapeutic efficacy of ^225^Ac-PSMA-617 involved two patients with late-stage, metastatic, castration-resistant prostate cancer (mCRPC) and complete remissions [59]. Additional clinical studies have further revealed the remarkable anti-tumor activity and the promising duration of tumor-control of treatment with ^225^Ac-PSMA-617 [6,59,60]. These studies have further demonstrated the significant potential of TAT (Table 3, Figure 1). Xerostomia (dry mouth syndrome) was the main side effect, indicating that further modifications in clinical trial design might be necessary to further enhance the therapeutic range.

### 4.2. Peptides

Peptides are oligomers of amino acids that may sometimes exhibit secondary structure; they may have branched or linear frameworks, and may be composed of varying amounts of non-canonical monomers. The polypeptide chains of peptides can have anywhere from 2 to 70 amino acids but more typical examples of targeting peptides are made of less than 10–15 amino acids (1000–1500 MW). Owing to this molecular weight and their capacity to be synthesized and modified with conventional organic synthesis techniques, peptides have long been utilized as targeting agents for radionuclide therapies and diagnostic applications. 

**Figure 4:**
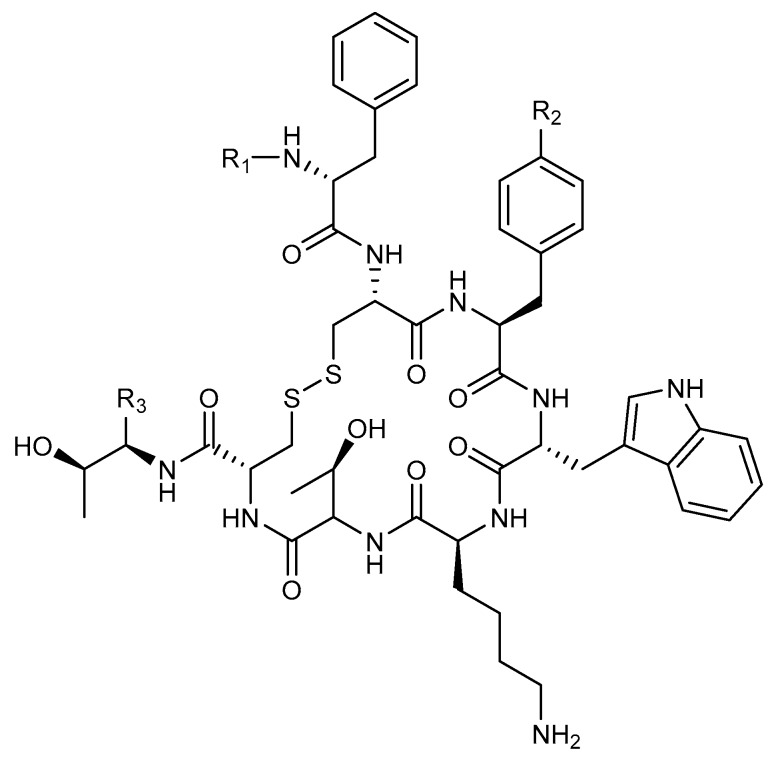
The somatostatin mimetic octreotide and its DOTA-containing analogs. Octreotide: R_1_ = H, R_2_ = H and R_3_ = CH_2_OH; DOTATOC (endotreotide): R_1_ = DOTA, R_2_ = OH and R_3_ = CO_2_H; DOTATATE (Octreotate): R_1_ = DOTA, R_2_ = OH and R_3_ = CH_2_OH.

Since the 1980s, analogs of the endogenous peptide hormone somatostatin have been developed as therapeutics for neuroendocrine disorders [61]. The FDA approved octreotide (OC, Figure 4), the cyclic octapeptide, upon which much of the early peptide receptor radionuclide therapy (PRRT) was based. Chelating molecules attached and various radionuclides for β and α-emission therapies have been reported. Figure 4 details the structure of OC and two of its commonly-used PRRT ligand analogs, DOTATOC and DOTATATE. 

Nephrotoxicity is often one of the highest concerns in PRRT due to tubular reabsorption of the labeled peptide after glomerular filtration [62]. Seventy-four human patients were followed (>1 year) after PRRT with the β-emitter ^177^Lu-Octreotate. Renal function was monitored by accessing glomerular filtration rate (GFR). Somewhat surprisingly, renal impairment was minimal, with only 43% of patients experiencing a decrease in GFR (<2 mL/min/m2 per year) and 11 patients actually saw increased GFR (>10 mL/min/m2 per year) [63]. The first published account of the α-emitter ^213^Bi in an OC analog (DOTATOC, Figure 4) was reported in 2006. The radio-peptide ligand was shown to retain its affinity for the somatostatin receptor and inhibited tumor growth in a somatostatin receptor-positive rat pancreatic tumor model (CA20948) [64]. Importantly, the study followed major organ toxicities in rodents. Little to no nephrotoxicity was observed at the various administered activities (13–22.2 Mbq). 

More recently, even tumors that had become radioresistant to β-therapy (^90^Y and ^177^Lu–DOTATOC) responded to ^213^Bi–DOTATOC in a clinical model of eight human patients [65]. The treatment for each patient was individualized according to each particular disease state and each patient had substantially positive outcomes in terms of tumor regression and survival. Critically, the acute hematological toxicity normally associated with the analogous β-therapies was only moderate. This limited report suggested only a mild reduction in acute renal function. No other major acute toxicities were reported. This first-in-human report of this peptide-targeted alpha therapeutic may lay the ground work for future human TAT using peptide-targeted systems.

It has been the common supposition that TAT would work well for small, disseminated tumors but not as well for large, solid tumors. Recently, DOTATATE (Figure 4) was labeled with ^213^Bi and used to investigate efficacy differences in tumor size among two tumor models, rat pancreatic (CA20948) and human small cell carcinoma (H69) with each containing a small (50 mm^3^) and large (200 mm^3^) tumor group [66]. Both tumor lines maintained high expression of SSTR_2_ and all tumor groups saw delayed tumor growth and higher survival over controls. Three mice from the smaller tumor groups were effectively put into remission through the end of the study. This report has implications for TAT in regard to tumor receptor heterogeneity, tumor size and perfusion. More recently, α-emitting radionuclides have been conjugated to melanocortin 1 receptor targeting peptides and efficacy determined in the pre-clinical treatment of mouse xenograft models of cutaneous and uveal melanomas [67,68,69].

### 4.3. Antibodies

Full length immunoglobulins (IgGs) are typically in the 150 kDa molecular weight range, and have been proven to have high binding affinity and specificity to a broad range of extracellular receptors. Developments in hybridoma cell line technology have opened the door to the production of monoclonal antibodies (mAbs) which can be labeled with chelating molecules, to which radionuclides can be added. This approach to specifically deliver ionizing radiation payloads is termed radioimmunotherapy (RIT). While many of the examples here will involve radioactive payloads directly conjugated to the protein, antibodies have also been employed to target macromolecular payloads, such as nanoparticles and liposomes, to cellular targets [70,71,72]. 

A research group based in the Memorial Sloan-Kettering Cancer Center (MSKCC) has reported several accounts of their work labeling trastuzumab with actinium 225. This mAb is the well-known and FDA approved *HER2/ERRB2*-targeting agent. Initially, they showed through a three cell line, spheroid in vitro model that their α-RIT scheme could penetrate spheroids, retard growth and prevent regrowth of colonies in a dose dependent manner [73]. While a promising start, this work underscored the importance of target expression and also suggested challenges of RIT due to extravasation of targeting agents in normal tissues and toxicities of released/free decaying daughter products [74,75]. 

The MSKCC Scheinberg group’s work eventually led to the first clinical trial of an actinium 225 chelated targeting antibody (Clinical Trials.gov Identifier: NCT00672165). This α-RIT scheme utilized the previously-explored humanized anti CD33 antibody, limtuzumab, to target acute myeloid leukemia cells. Subsequently, that preliminary work led to the birth of Actinium Pharmaceuticals, Inc. and a portfolio of targeted ^225^Ac conjugated constructs in both preclinical and clinical pipelines. 

**Figure 5:**
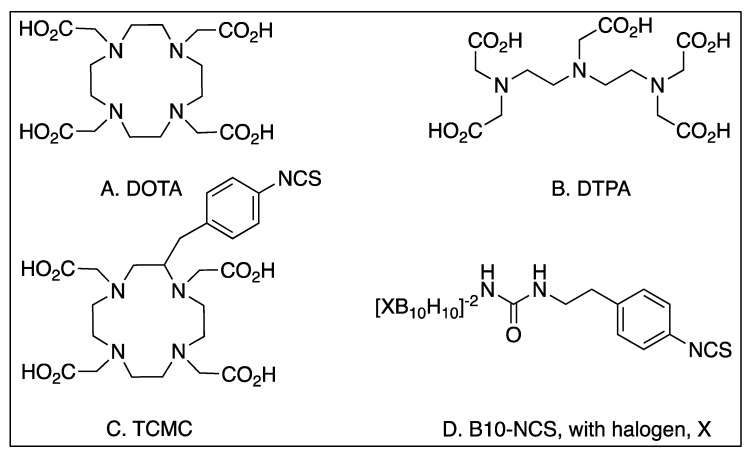
Common metal chelators and binders used to attach radionuclides to targeting ligands.

Astatine-211 has also been utilized for α-RIT. In 2013, Orozco and coworkers coupled a decaborate cage structure (B10, Figure 5) with ^211^At to anti-CD45 antibody in an attempt to target acute myeloid leukemia [76]. More recently Green and coworkers reported an example using anti-CD20 mAb conjugated with a ^211^At with a similar B10 labeling scheme [77]. This study sought to eliminate minimal residual disease (MRD) in a mantle cell lymphoma animal model. Their remarkable results showed 70% eradication of the MRD in animals bearing a disseminated model. However, the subcutaneous lymphoma xenograft group treated with the same agents only saw prolonged survival (two to three-fold) without any cured animals. This study is clear evidence of the need for tumor perfusion of the TAT. 

### 4.4. Antibody Fragments

A limitation of full-size IgGs is their typical 1–3 week serum stability. While this kind of circulation may be advantageous for certain therapeutic applications, for radioimmunotherapy (RIT) it presents a serious liability. Excess antibody can continue to circulate, lowering the tumor-to-nontumor (T/NT) ratio, particularly with respect to the tumor-to-blood (T/B) ratio. Finally, high levels of continuously-circulating α-emitters result in hematological toxicities, as well as extravasation in normal tissues. 

In attempts to shorten the long plasma half-life of full-length antibodies, many groups have sought to reduce the sizes of the mAbs while retaining their remarkable binding characteristics. To those ends, antibodies have been cleaved into smaller sections through enzymatic digestion and also engineered ab initio [78,79]. For comparison, the engineered antibody fragments of diabodies (Db) and minibodies (Mb) have typical circulating half-lives of 2–5 and 5–12 h, respectively, while the circulating half-life of full-length IgGs is more typically 1–3 weeks. Monovalent and bivalent affibodies (7 and 15 kDa respectively) have been developed with targeting specificity to *HER-2* and they have plasma half-lives of roughly 45 min [80]. The science of antibodies is ever progressing and new classes are still being observed in nature; nanobodies (13–14 kDa) are heavy chain-only antibodies, that have been isolated from *Camelidae* and have lately been conjugated with theranostic radionuclides [81]. These new categories of targeting constructs are adding to the growing body of evidence that there may be a so-called ‘Goldilocks’ zone in terms of the size of targeting constructs.

### 4.5. Passive Targeting

Another strategy often employed for TAT is based on the natural selective uptake and accumulation of certain elements. For instance, the actively restructuring environment of osteoblastic bone metastases is conducive to calcium accumulation in the form of hydroxyapatite. Due to the resemblance of radium-223 to calcium, it can act as a surrogate and be incorporated into the rapidly-growing matrix [7,29]. Indeed, the recent approval of ^223^Ra dichloride therapies for metastatic castrate-resistant prostate cancer has been followed in patients for over twelve months with moderately successful outcomes [82]. Other studies have looked to ^223^Ra dichloride for the treatment of the bone metastases from hormone-refractory breast cancer [83,84].

The enhanced permeability and retention (EPR) effect is often regarded as a rationale for the passive targeting of large macromolecules like nanoparticles and liposomes. This accumulation of macromolecules through the leaky vasculature of quickly-dividing tumor cells also provides the opportunity to incorporate multiple radionuclides into the framework of a targeting molecule. Additionally, it has been proposed that nanoparticles or liposomes could be used to sequester decaying daughter products that would otherwise be released to freely circulate in the blood because of damage to molecular bonds due to α-decay or metabolism of the radiopharmaceutical [85,86]. 

## 5. Chelation/Attachment

### 5.1. Radiosynthesis

The recent approval of ^223^RaCl_2_ has reinvigorated research into TAT development [87]. Several α-particle-emitting radionuclides have been described in the literature, and these include ^211^At, ^212/213^Bi, ^212^Pb and ^225^Ac [44,88,89]. With the exception of ^211^At [41,42,90], all are radiometals that require a bifunctional chelator for the attachment of the therapeutic radioisotope to the targeting moiety. The chemistries of ^212/213^Bi and ^212^Pb have been covered extensively elsewhere [91,92,93,94]. The radiochemistry of radium has recently been reviewed [95]. This section will cover recent advances in ^225^Ac chelation chemistry that have occurred since the publication of the excellent review by Scheinberg and McDevitt in 2011 [96]. 

^225^Ac, whose decay chain was not finally resolved until the middle of the 20th century is one of 29 radioactive isotopes of actinium, which is the first element of the actinide series [97]. Although the lack of non-radioactive actinium isotopes has hindered the exploration of ^225^Ac chemistry, the pioneering work of Seaborg and others has provided an important understanding of the basic reaction chemistry of this element [97,98,99]. Actinium chemistry has been observed to closely follow that of lanthanum chemistry, since both exist as trivalent ions in solution. These observations have led to the belief that La^3+^ can be an important non-radioactive surrogate for Ac^3+^, with the caveat that the differences in ionic radii must be considered carefully [100]. 

Despite the relatively limited knowledge of actinium chemistry, the medical community has seized upon its therapeutic potential in TAT. Using lanthanide chemistry as a guide, many attempts to attach this radioisotope to targeting molecules have been reported with a variety of acyclic and cyclic ligands that would effectively chelate this radioisotope [101,102,103,104,105,106]. To date, however, only 1,4,7,10-tetraazacyclododecane-1,4,7,10-tetraacetic acid (DOTA) has found widespread acceptance as an ^225^Ac chelator in both preclinical and clinical applications [96], despite the low radiochemical yields and specific activities that have been reported [73,104,107]. Maguire et al. have described an improved, one-step ^225^Ac radiolabeling procedure of monoclonal antibodies that resulted in a 10-fold increase in radiolabeling yield and a 30-fold increase in specific activity [108]. The authors concluded that the improvements in yield and specific activity resulted from greater control over the DOTA–mAb conjugation reaction and the improved chelation of ^225^Ac during radiosynthesis. Additionally, the new procedure did not alter antibody activity or therapeutic efficacy in vivo, which further suggests the usefulness of this strategy. More recently, Poty et al. reported a single-step click chemistry approach to ^225^Ac-radioimmunoconjugate synthesis [109]. The click approach generated superior radiochemical yields compared to the standard two-step strategy, suitable specific activities, good in vitro stability and excellent biodistribution profiles. The authors proposed that said novel click strategy could be applied when the targeting vector is unstable under the conditions used for the standard method.

Despite improving the radiochemical yield and specific activity of ^225^Ac radiopharmaceuticals, important challenges, which include minimizing the detrimental effects of recoil energy and effectively sequestering the cytotoxic daughter products ^221^Fr and ^213^Bi after ^225^Ac decay, remain to be overcome. While several reports have described cellular internalization as an effective method of sequestering ^225^Ac and its daughter products [35], scientists are increasingly turning to nanotechnology to resolve this issue, and a variety of nanoparticles, including liposomes and carbon-based nanoparticles, have been explored [96,110,111,112,113,114,115]. Recent reports have demonstrated that LaPO_4_ nanoparticles hold promise as safe and effective nanocarriers for ^225^Ac [71]. In these studies, the authors synthesized LaPO_4_ (monazite) nanoparticles doped with ^225^Ac. These particles were prepared with functionalized surfaces that facilitated the attachment of 201B mAb, which targets murine thrombomodulin on lung epithelium. Upon in vivo injection, targeted particles accumulated rapidly in the lung through specific interactions between the 201B mAb and its antigen. Moreover, retention of ^225^Ac and half of the ^221^Fr and ^213^Bi daughter nuclei within the particles demonstrated the potential for trapping radionuclides after high energy emissions. McLaughlin et al. improved upon this work by examining ^225^Ac-doped LaPO_4_ nanoparticles that were encapsulated by GdPO_4_ and Au shells [86]. These particles had an average diameter of 27 nm, and were easily conjugated to the 201b mAb using standard bioconjugation techniques. Biodistribution and small animal single-photon emission computed tomography (SPECT) studies revealed the specific targeting of murine thrombomodulin within the lung epithelium that was reduced upon the administration of non-radioactive 201b mAb. Interestingly, the multi-layered nanoparticle design seemed to increase the retention of ^225^Ac, ^221^Fr and ^213^Bi within the particle, which reduced the radiation dose experienced by the kidney. Approximately four and five-fold reductions were observed at 1 and 24 h post-injection, respectively. These data demonstrate that multi-functional, layered nanoparticles have the potential to deliver and retain ^225^Ac and its daughter radionuclides at the target site, while minimizing off-target toxicities that can occur from errant daughter products in the ^225^Ac decay chain. 

### 5.2. Linkers/Rational Design

The most utilized chelating molecules for targeted radionuclide therapies using biological molecules are 1,4,7,10-tetraazacyclododecane-1,4,7,10-tetraacetic acid (DOTA) and diethylene triamine pentaacetic acid (DTPA), and their related analogs (Figure 5). Depending on the how the compound is linked to the targeting ligand, these organic structures feature 3 or 4 carboxylates that are negatively charged at physiological pH, and along with the lone pair electrons from each of the 3 or 4 nitrogens, they can coordinate metal ions like α and β-emitting radionuclides. Often, these chelating molecules are linked to the targeting molecule by forming a new amide bond between an amine on the targeting molecule and a carboxylate on the DOTA/DTPA. This strategy is particularly advantageous in the case of peptide synthesis since the reaction, characterization and purification of the linker addition can be part of the overall synthesis of the targeting ligand. 

**Figure 6:**
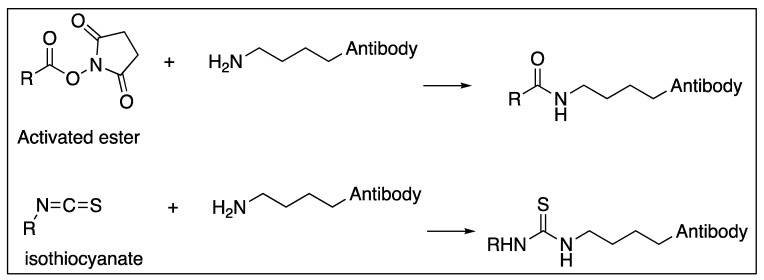
Common conjugation chemistries used to functionalize biomolecules. Countless other schemes have been well characterized and the reader is directed to Bioconjugates Techniques for a review and protocols [117].

When DOTA or DTPA are linked to proteins like antibodies, amide bonds are typically created between a primary amine of surface-exposed lysine residue and an activated carboxylate on the DOTA or DTPA (Figure 6). Another widespread chemistry available to link protein lysines with DOTA or DTPA is through an isothiocyanate which yields a stable thiourea (Figure 6) [53,116]. While these newly formed amide bonds create the same covalent structure as the peptide-synthesized version, there are important distinctions. In the stochastic reaction of any accessible lysine, many different combinations are possible, and there is little to no control over the regioselectivity of the conjugation. It is unlikely, but possible, that targeting ligands could block or hinder the antigen-binding sites on the antibody. 

The importance of site-specific modifications to biologic targeting motifs is only recently becoming clear. In a 2014 paper from UCLA [118], diabodies (Dbs) were conjugated both site-specifically through reduced cysteines and non-specifically through accessible lysine ε-amines to DOTA chelators. While the tumor-to-blood ratio of the specifically labeled protein was moderately higher than the more heterogeneous product, the more striking result was the renal and hepatic distribution. Kidney uptake levels were almost doubled for the cysteine-labeled Db, and liver uptake levels were reduced for the non-specific amine-labeled Dbs. 

Other important research regarding linkers has uncovered different methods to reduce kidney toxicity, via the renal reabsorption of radiolabeled peptides and antibody fragments, as they are filtered by the glomerulus. By taking advantage of renal brush border enzymes, the radionuclide can be cleaved from targeting ligand and excreted. By engineering an antibody fragment with a C-terminal lysine, and subsequently modifying the ε-amine with DOTA:indium-111, Li and coworkers demonstrated a 50%-60% reduction in the kidney-uptake of the radionuclide [119]. More recently, a Japanese group probed the brush border enzymes to understand more about their specificity and they were able to pinpoint a glycine-tyrosine linkage that specifically cleaved a radio-iodinated benzoate derivative from an antibody Fab fragment [120]. Presumably, a similar strategy could be used for the α-emitting halogen, ^211^At. 

Polyethylene glycol (PEG) linkers are made of repeating –CH_2_CH_2_O– monomers and are commonly employed by chemists to alter biodistribution and pharmacokinetics. PEGs can be prepared in two categories, polymeric and discrete oligomers. Bifunctionalized versions of these polymers can link targeting molecules with the chelating agent or just add bulk/solubility to lower molecular weight entities. Researchers wishing to reduce immunologic response to nanoparticles have been known to decorate their macromolecules with PEG [70], as was the case with McLaughlin and coworkers with PEG12 linker used to link ^225^Ac containing nanoparticles to mAb [86].

## 6. Radiation Dosimetry

Dosimetry provides means for evaluating the efficacy of a radiation therapy modality [3]. Because the goal is to a deliver high dose to tumor cells with a minimal dose to normal tissues, it is of great importance to quantify accurately where, when and how a dose is being deposited. The Medical Internal Radiation Dose Committee (MIRD) developed an approach to determine the average absorbed dose from internal radionuclides [121]. The absorbed dose method accounts for low LET radiation where thousands of statistically independent deposition events across a cell are required to induce a biological effect [3]. With respect to an α-particle’s interactions, the averaged absorbed dose will fail to characterize biological outcome. Because the α-particle range is only a few cell lengths, some cell nuclei will receive multiple traversals while others will receive zero traversals. Additionally, the location of the α-particle track will determine the energy absorption by the cell. These factors result in large statistical variability of energy deposition and stochastic effects become important. To account for those effects, Roeske developed a microdosimetric methodology based on the mean dose to target cells, probability distribution of specific energy absorbed by target cells, and the fraction of cells receiving zero hits [122]. Kellerer and Chmelevsky described the requirement that microdosimetry must be used when the relative deviation of local dose is greater than 20% [122]. Following this criterion, microdosimetric techniques are more important in analyzing non targeted cells where the local average dose is small, resulting in larger variations than targeted cells. 

In most cases, the activity level and mean absorbed doses to the target are large, resulting in a low expected stochastic deviation, and therefore, there is no need to use microdosimetric techniques. The MIRD Pamphlet No. 22: Radiobiology and Dosimetry of Alpha-Particle Emitters for Targeted Radionuclide Therapy recommends using the conventional MIRD formalism [3]. The mean absorbed dose to the target region, $r_{T}$, from a source region, $r_{S}$, due to a particular emission type, *x*, over a dose integration period, $T_{D}$, is given by:

(1) $$D_{x}\left(r_{T},T_{D}\right)=\tilde{A}\left(r_{S},T_{D}\right)\frac{\sum_{i}\Delta_{i}^{x}\varphi \left(r_{T}\leftarrow r_{S};E_{i}^{x}\right)}{M\left(r_{T}\right)}$$

where $\tilde{A}\left(r_{S},T_{D}\right)$ is the total number of nuclear transitions in the target region; $\Delta_{i}^{x}$ is the mean energy emitted per disintegration for the *i*-th emission of type *x*; $\varphi \left(r_{T}\leftarrow r_{S};E_{i}^{x}\right)$ is the fraction of energy emitted per nuclear transition in the source region that is absorbed in the target region by the *i*-th emission of type *x* that is emitted with initial energy *E*; and $M\left(r_{T}\right)$ is the mass of the target region. The total number of nuclear transitions is determined by measuring activity levels in tissue samples at several points in time post administration. The activity levels plotted against time gives a time-activity curve which is integrated to obtain the total number of nuclear transitions. The mean energy emitted per emission $\Delta_{i}$ is a physical property of the radionuclide and can be obtained from nuclear decay tables. The absorbed fraction for each decay type is dependent on the reference phantom geometry and obtained through Monte Carlo calculation. The dose contribution from each emission type is then weighted by its relative biological effectiveness, and then summed:

(2) $$D_{RBE}\left(r_{T},T_{D}\right)=RBE_{\alpha }D_{\alpha }\left(r_{T},T_{D}\right)+RBE_{e}D_{e}\left(r_{T},T_{D}\right)+RBE_{ph}D_{ph}\left(r_{T},T_{D}\right)$$

Several assumptions are made when using this formalism of dose calculation. It is assumed that the activity is uniformly distributed in the organ and that α-energy deposition is also uniformly distributed in the organ. It is also assumed that dose from both α and β-emissions are locally deposited. The calculation of the absorbed fractions is based on idealized phantoms that have standard geometries which cannot account for the individual anatomy of a patient. It is also important to note that radioactive daughters are not taken into account. Therefore, daughter decays must also be calculated, as well as accounting for daughters’ biodistribution. The formalism was developed as an adequate method for the dosimetry of internal radionuclides used for diagnostic purposes and lacks the accuracy needed for therapeutic applications. 

As a first method to improve on the original MIRD formalism, phantoms were developed to better match the standard human anatomy. For example, Christy and Eckerman developed phantoms that represented a male, a female and children [123]. Later, as computational power increased, voxelized phantoms like VoxelMan were created based off 3D imaging [124]. MIRD Pamphlet No. 17 describes a method to extend the S value formalism to the voxel level to account for nonuniform distributions of activity [125]. The most extensive software that has adapted the MIRD S factors is OLINDA/EXM, which has calculated an internal dose for over 200 radionuclides, including α-emitters, in 10 different phantoms [126].

The voxel S method is still a model-based approach to dosimetry. There is much less tolerance for inaccuracies in therapeutic applications, which calls upon the need for patient-specific dosimetry. In order to accomplish this, the patient’s own anatomy, obtained from CT, is used in combination with SPECT imaging to obtain 3D spatial distribution of activity. It is advantageous for the α-emitting radionuclide to also have γ photon emission as it decays so that SPECT imaging can provide information about regional uptake. The method to calculate a 3D, imaged-based dose is as follows [127]. Serial SPECT studies taken over time post administration of the radiopharmaceutical can determine the pharmacokinetic variations within an organ on the voxel level. The SPECT/CT images are registered together and integrated voxel by voxel over time to obtain the 3D, time-integrated activity. The CT provides a map of tissue electron density for each voxel, which is necessary for dosage calculations. A Monte Carlo package is then employed using the activity distribution as the source definition and energy deposition is tallied in each voxel. Several software packages are available for 3D, patient-specific dosimetry, including MCID [128], OEDIPE [129] and SCMS [130] which are based on MCNP, 3D-RD [131] and DOSIMG [132] which are based on EGS [133], RAYDOSE [134] and RAPID [135] which are based on GEANT4 [136], and DPM [137], which is not a public domain code. Both MCNP and GEANT4 can simulate α-particles, while EGS can only transport photons and electrons.

While imaging-based dosimetry can account for non-uniformity and calculate dose at the voxel level, it is still a macroscopic quantity. The spatial resolution of current SPECT imaging ranges from 5 to 25 mm [31]. These dimensions are much larger than the 40–90 µm range of an α-particle. This results in the stochastic variations due to α-particle energy depositions not being taken into account and the voxel dose could be misleading. For α-particles, the ideal dosimetric targets are isolated cancer cells in transit in the vascular and lymphatic systems, micro-metastases and tumor capillary networks [31]. The only way to study the dosimetry of α-particles on a small scale is pre-clinically. These have been limited to in vitro measurements and microdosimetric Monte Carlo models. However, recently, several groups have developed alpha cameras, such as the Ionizing-Radiation Quantum Imaging Detector, which provides images of the ex vivo activity distribution with a spatial resolution on the order of tens of microns µm [138,139]. Monte Carlo methods can then be employed to calculate the dose absorbed on that scale. This is viewed as an important advancement in alpha dosimetry because no further modeling was needed to obtain the spatial and temporal activity distribution. 

## 7. Pre-Clinical Studies

### 7.1. Preclinical Therapeutics Studies

In recent years, a number of pre-clinical studies have been reported that provide strong evidence of the potential for use of TAT in the treatment of cancers. See Table 2 for a summary of the published in vivo preclinical TAT studies (*n* = 48) that have used animal models of cancer. Most of these studies (81%) have involved the use of immunoconjugates where monoclonal antibodies serve both as the targeting moiety and as the attachment scaffold for the α-emitting radionuclide. Two of these immunoconjugates involved the use of antibody fragments (scFv) [140,141] and one used a diabody [142]. Three studies involved pretargeting, where a cancer targeting antibody–streptavidin construct, or antibody–TCO (*trans*-cyclooctene) construct for click chemistry, was injected prior to delivery of an α-emitting radionuclide attached to biotin [141,143,144]. In this study, pre-targeting demonstrated increased efficacy over TAT using the targeting antibody alone. Peptide-based targeting moieties were used in 15% of these studies. In other individual studies, a protein [145], a small molecule ligand [146] and an inorganic compound [147] were used as targeting groups. 

As is the case for most radioimmunotherapies in current use, that typically deliver β-emitting radionuclides, a number of the reported TAT studies (23%) targeted non-solid tumors; i.e., leukemias, lymphomas and multiple myeloma. A majority of these non-solid tumor studies reported significant efficacy and/or decreased systemic toxicity relative to controls. Since α-emissions have higher LET over a shorter range, there is significant interest in the use of TAT for treatment of solid tumors. This is evident in the preclinical studies reported, as 79% involved targeting of solid tumors and nearly all of these studies reported significant efficacy against solid tumors relative to controls, with nearly half also reporting low or manageable systemic toxicity. Four of these studies demonstrated increased efficacy using TAT relative to targeted beta (β)-emission therapy (TBT) using the same targeting moiety and tumor model [143,151,152,163,164,165,166,180,185].

### 7.2. Preclinical Imaging

Whether developing a small molecule or antibody-based therapeutic, pharmaceutical companies traditionally rely upon molecular imaging to assist them in identifying the most promising molecules in the research pipeline to be carried forward into clinical trials [203,204,205,206]. Furthermore, this strategy has been particularly effective in developing targeted, systemic β-based radiotherapies when the β-emitting radioisotope can be paired with a diagnostic surrogate for SPECT or PET imaging. Several of these pairs include ^64/67^Cu, ^86/90^Y and ^124/131^I [207]. However, with the exception of the ^203/212^Pb system [68,191,208], researchers developing TATs with other α-emitters such as ^213^Bi, ^211^At and ^225^Ac, have not been able to capitalize on this strategy. This limitation has hindered TAT development, but also stimulated attempts by the scientific community to observe the pharmacokinetics of TAT therapies using the decay of TAT radioisotopes in preclinical or clinical settings. For example, Sgouros et al. attempted to use the 440 keV γ emission of ^213^Bi to monitor the biodistribution of ^213^Bi-HuM195 in patients with leukemia [209]. Due to the short half-life of the radioisotope, data acquisition was limited to sixty minutes after patient injection, but this was sufficient to detect elevated levels of radioactivity in the red marrow, liver and spleen. While the data suggested that it would be possible to derive important pharmacokinetic and dosimetric data from the in vivo imaging of the ^213^Bi emissions, the large amount of activity necessary for imaging has precluded its routine use in a clinical setting. 

The decay scheme and extreme cytocidal potency of ^225^Ac further complicates the TAT community’s ability to develop preclinical and clinical imaging methodologies to monitor pharmacokinetics and estimate dosimetry. Since the 440 keV emission derived from ^213^Bi decay is considered too energetic for preclinical imaging applications, attention has focused on the less energetic 218 keV γ ray emission of ^221^Fr, which has been used to monitor the biodistribution of ^225^Ac-doped nanoparticles in mice [71,86], and this approach has shown promise. However, in this imaging paradigm, animals are typically euthanized 1 h after receiving the radiopharmaceutical and imaged 24 h after euthanasia to ensure isotopic equilibrium. Hence, these experiments do not address the potential of longitudinal SPECT studies to inform development of ^225^Ac-radiopharmaceuticals. Clearly, new approaches are needed, since the ability to image and visualize ^225^Ac-based radiopharmaceutical biodistribution, metabolism, and clearance in animal models through longitudinal imaging studies would be advantageous [210,211]. 

Cerenkov luminescence imaging has emerged as an alternative to traditional nuclear medicine techniques for visualizing the delivery and biodistribution of TATs. Cerenkov luminescence is derived from the emission of ultraviolet light when certain charged particles exceed the phase velocity of light in a given medium [212,213], and this effect can be observed using standard optical imaging systems originally designed to detect bioluminescence and fluorescence. Recently, Cerenkov emissions were observed with the decay of several medically relevant isotopes, including ^225^Ac, which was reported to yield the largest optical signal among all isotopes examined [211,214]. Alpha (α) particles however, do not travel with sufficient velocity to generate Cerenkov emissions, which led researchers to postulate that the emissions observed resulted from the β-decay of the ^213^Bi, ^209^Tl and ^209^Pb daughter products. Subsequent publications, using theoretical and experimental means, described the association between Cerenkov radiation and ^225^Ac decay [214,215]. While both research groups determined that Cerenkov luminescence imaging (CLI) could be accomplished with ^225^Ac and other α-particle emitting radionuclides, it was determined that radionuclides’ decays solely by α-particle emission do not produce sufficient Cerenkov radiation to be useful for imaging. Secondly, a time delay would be needed for equilibrium to be established between the parent radionuclide and its daughter products. For ^225^Ac, this delay was determined to be ten hours. This precludes the imaging of small molecules and peptides at early time points, but allows one to monitor the biodistribution of ^225^Ac-labeled antibodies and nanoparticles, which require extended circulation time for effective tumor targeting and blood clearance. Finally, the high recoil energy associated with ^225^Ac and daughter product release from the original conjugate must be considered. The emission of Cerenkov radiation may not reflect the actual biodistribution of the radiopharmaceutical in question, but of the daughter products producing the Cerenkov emissions [216]. Sequestering the daughter products with the original conjugate is important if this technique is to provide any benefit to the TAT community. In proof of principle studies, Pandya and coworkers seized upon these recommendations and further tested the association between ^225^Ac decay and Cerenkov radiation in living animals [217]. The authors synthesized ^225^Ac–DOTA–c(RGDyK) and evaluated its stability, biodistribution and potential use as an imaging agent in CLI in a murine model expressing human glioblastoma U87mg tumors, which overexpress the α_v_β_3_ integrin. Additionally, they exploited the well-documented ability of RGD-containing ligands to be internalized on integrin binding to sequester the ^225^Ac and the daughter products within the tumor. This technique, often referred to as the nanogenerator approach, has been used successfully to sequester ^225^Ac-based radiotherapeutics and daughter decay products within a cell to increase their therapeutic effectiveness [35,149,150]. Surprisingly, all animals demonstrated no signs of distress during these experiments. When compared to α_v_β_3_^-^ tissues, in vivo CLI revealed five-fold more luminescence from the α_v_β_3_^+^ tumors. Luminescence was also observed in the liver and kidney tissues, which have been shown in related biodistribution studies to be involved in the clearance and excretion of this radiopharmaceutical [218,219,220]. Ex vivo image analysis also revealed a similar trend as the in vivo results, and the addition of a blockade reduced the luminescence emitted from the tumors by 80%, which suggested to the authors that activity delivered by the radiopharmaceutical and internalized through specific receptor-interactions was being retained in the tumor. To further validate the theranostic potential of their approach, they also performed preliminary therapy studies in U87mg tumor-bearing mice, which illustrated the cytocidal potency of their α-emitting radiotherapeutic [221]. While more studies are needed, these promising results suggest that this area of research may have broader implications for molecular imaging and may help to facilitate TAT development. 

## 8. Medicinal Chemistry

### 8.1. Lead Optimization

As previously touched upon, the characteristics of various targeting ligands, like lipophilicity, molecular weight and ionization potential are all critical variables that help modulate radiopharmaceutical properties. For example, small, polar compounds may undergo rapid renal excretion. The addition of a PEG linker will add water solubility, bulk, and may help avoid kidney toxicities. Conversely, the removal of an ionizable group (e.g., sulfonate or protonated amines at physiologic pH) during medicinal chemistry design may make the targeting ligand more hydrophobic and shift clearance toward initial liver processing. Therefore, when working to reduce targeting ligand complexity or increase the ease of synthesis, these physical attributes of the compound must be taken into consideration.

Antibodies and engineered fragments can also benefit from medicinal chemistry optimization. Many chelators and linkers covalently attached to antibodies and their fragments produce heterogeneous products. In the most typical fashion of linker attachment, surface accessible lysine side chains are utilized to form covalent bonds with radionuclide chelating molecules (Figure 6). Site-specific antibody modifications can yield more homogeneous products. The Rader group at Scripps Florida has made such progress by engineering a selenocysteine residue into targeting antibodies, allowing for complete control of regiochemistry during functionalization [222].

Another route to increasing T/NT ratios is through the notion of pretargeting. These strategies operate under the premise that nonradiolabeled, bifunctional targeting agents can be administered to the patient and allowed to converge to their target. Once the unbound portion has cleared, a fraction of radiolabeled molecule with a binding affinity for the bifunctional targeting agent is administered. Typically, the radiolabeled agent is designed to clear quickly if unavailable for binding; thus, lowering off-target radiation. Typical binding partners amenable to pretargeting schemes include avidin/streptavadin–biotin, DNA–DNA or antibody–hapten [223]. For a more in-depth analysis of pretargeting strategies please see the recent review article from Frampas and coworkers [224].

Novel combination therapies are also now being investigated. Combination therapies can be particularly useful in heterogeneous diseases like multiple myeloma. Anti CD138 mAb conjugated with ^213^Bi has been tested synergistically with the chemotherapeutic melphalan to help eliminate residual disease in a multiple myeloma model [116]. Interestingly, this study saw no benefit to the combination therapy when compared with RIT alone. The speculation was that the follow-up treatment with CD138-targeted mAb was less successful due a swift reduction in CD138 antigen in cells progressing through apoptosis [225], illustrating the importance of the orthogonality in study design as it relates to combination treatments and target expression.

### 8.2. cGMP Production

Current good manufacturing practice (cGMP) standards for the production of the targeting ligand precursors before the incorporation of the α-emitting radioisotopes are similar to those used for the manufacturing of peptide, antibody or antibody fragment drug and drug candidates. There are numerous cGMP-compliant peptide synthesis companies, and often the preclinical grade peptides will be prepared by standard solid-phase synthesis and purified to ≥ 95% purity, but cGMP-grade peptides will often be prepared via routes that allow greater control over the purity and reliability of the synthesis. For instance, cGMP peptides are more likely to be prepared using a greater reliance on solution phase-synthesis procedures than preclinical grade peptides because the costs can be much lower on scaled-up batches of peptide, and more importantly, impurities can be removed as they build up during a solution-phase synthesis, whereas impurities just build up during standard solid-phase synthesis procedures. The recent development of a fully solid-phase synthesis of cGMP-quality Fuzeon^®^ is an exception to this trend that may lead to a greater reliance on fully solid-phase synthesis procedures for the production of cGMP peptides. Frequently, solid phase methods are used in combination with solution phase synthesis methods. As an example, preclinical grade linear peptides can be prepared and cyclized to the final product on resin, whereas cGMP grade peptides will often be prepared on resin and purified and then cyclized in solution, due to the easier analysis of reactions in solution. 

The cGMP production of antibody and antibody fragments will be focused on for the development of robust methods of production often using hybridoma technologies and purification by at least two orthogonal separation methods, usually involving an affinity column purification step followed by a size exclusion type purification system. Often these biotechnology-based manufacturing methods produce antibody or antibody fragments that are chemically programmed to provide specific sites for metal chelate attachment. For a recent review of this topic please, see the two-part article from Adumeau et al. [226,227]. 

The results of these manufacturing processes are lyophilized powders or relatively-concentrated, sterile, aqueous buffer solutions of the targeting moieties that are ready to chelate the α-emitters. The main difference between cGMP radiosynthesis for clinical trials and research grade radiosynthesis in the United States is the implementation of an FDA-compliant audit trail. Since the development of α-emitting radiopharmaceuticals is happening globally, another major issue concerning production and distribution of these novel therapies is the consideration of the local regulatory environment. A discussion of the potential for harmonization of the various international regulations has recently been published [228].

## 9. Clinical Studies

### 9.1. Recent TAT Clinical Trials

Despite the potential therapeutic benefits of the α-particle emitting radioisotopes over β-particle emitters, the restricted availability of relevant radionuclides and limited number of phase III clinical trials has so far limited the clinical use of TAT. Table 3 summarizes the TAT clinical studies conducted to date. Clinical trials prior to 2014 were reviewed by Elgqvist et al. [34] and we will focus on the more recent trials herein. The use of short-lived α-emitters like ^211^At (T_1/2_ = 7.2 h), ^212^Bi (T_1/2_ = 60 min) and ^212^Pb (T_1/2_ = 10.6 h) has been subdued so far, due to production and distribution limitations. However, this is not the case for ^213^Bi (T_1/2_ = 46 min), which, going back over the past two decades, has been used in the largest number of TAT clinical trials to date. Recent ^213^Bi studies have included the targeting of peptides for the treatment of glioblastoma [229,230] and neuroendocrine tumors [65], and the targeting of monoclonal antibodies for the treatment of bladder cancer [231,232]. Since the approval of ^223^Ra-dichloride for the treatment of CRPC in 2013, ^225^Ac has also emerged as a targeted therapeutic radionuclide of interest that has been used for targeting a small molecule [5,6,233], peptide [234] and monoclonal antibody [235] for the targeting of prostate cancer, neuroendocrine tumors, and acute myeloid leukemia (AML), respectively. Clinical studies of TATs using ^225^Ac or ^213^Bi as the therapeutic radionuclide have recently been reviewed [58]. Although the responses have been impressive for some, ^225^Ac–PSMA-617 in particular, the completion of more randomized clinical trials comparing TAT to standard of care will be essential to determine the clinical utility of these new therapeutic approaches. 

### 9.2. Radium-223 Dichloride (^223^RaCl_2_) Trials

In the following section, we will discuss the first and only clinically-approved TAT in the United States so far. After more than a 100 years since the discovery of the radium element in 1898 by Marie and Pierre Curie, on May 15, 2013, one of its radioisotopes, radium-223 (Ra-223) became the first α-emitting radionuclide to be approved by the Food and Drug Administration (FDA) in the form of radium-223 dichloride (^223^RaCl_2_, Xofigo^®^ formerly Alpharadin, made by Bayer HealthCare Pharmaceuticals Inc.) for the treatment of patients with castration-resistant prostate cancer (CRPC), with symptomatic bone metastases, and no known visceral metastatic disease [252]. This was based on the improvement in overall survival following intravenous administration of ^223^RaCl_2_ in patients with advanced prostate cancer with metastases to the bone, demonstrated in a double-blind randomized placebo-controlled trial called the ALSYMPCA (alpharadin in symptomatic prostate cancer) trial [7]. 

The role of systemic radionuclide therapy to the bone is to deliver therapeutically-effective radiation doses to multiple foci, including microscopic disease, as opposed to external beam radiotherapy, that gives more focal treatment and affects both normal bone marrow and metastases [253]. The two most commonly used radionuclide bone seeking agents for bone pain palliation are ^89^Sr (Metastron) and ^153^Sm-ethylene diamine *N,N*′-tetra(methylene) phosphonic acid (^153^Sm-EDTMP; Quadramet) [254], but these β-particle therapies, although effective for pain palliation, frequently cause myelosuppression and have not been shown to affect patient survival [255]. 

Cations of the alkaline earth elements (strontium, barium, and radium) have natural bone-seeking properties without the need for a carrier agent. As radium mimics calcium, when Ra-223 dichloride is injected intravenously, it forms complexes with the bone mineral hydroxyapatite at areas of increased metabolic bone activity, such as bone metastases, thereby exerting a highly localized antitumoral effect [7,256]. In addition, the Ra-223 daughter isotopes are also retained in the bone matrix [50]. The shorter penetration range of Ra-223 causes less bone marrow toxicity than bone-seeking β-particles [256]. The peak skeletal uptake of Ra-223 occurs within 1 h of injection, and blood radioactivity levels are <1% after 24 h with almost no redistribution of daughter nuclides from bone [256]. The total skeletal uptake ranges between 40% and 60% of the administered activity [257], and the excretion is predominantly via the gastrointestinal tract, with much less renal clearance [258]. Due to their significantly higher potency, the activities administered of α-emitters are much lower than β emitters, with significantly less radiation exposure to hospital staff and family members [259]. Merely basic hygiene measures and routine contamination protection barriers, such as latex gloves, are enough to protect both health care personnel involved in the patient’s radionuclide administration and the patient’s family. 

In the first-in-human single dosage phase I trial for patients with breast and prostate cancer skeletal metastases, single dose Ra-223 was well tolerated at all therapeutically relevant doses (46–250 kBq/kg intravenously [IV]) [257]. There was no dose-limiting hematological toxicity, only mild, transient myelosuppression (nadir 2 to 4 weeks after the injection) and mild transient diarrhea, with nausea and vomiting more frequently observed in the highest dosage group [257]. Subsequently, the phase I dose-escalation study in advanced prostate cancer with bone metastases, showed that repeated Ra-223 dosing was well tolerated to a total dose of 250 kBq/kg [50].

In the following phase II study of CRPC patients with symptomatic bone metastases, Ra-223 given at different doses (5, 25, 50 and 100 kBq/kg) was well tolerated with a positive effect on pain assessments (visual analogue scale [VAS] and analgesic use). At week 8, 56% and 71% of patients had reduced pain and stable analgesic consumption in the 50 and 100 kBq/kg groups, respectively. A statistically significant decrease in bone turnover markers, including alkaline phosphatase, was noted in the highest dose-group (*p* = 0.0067) [260].

The phase III, double-blind, randomized, placebo-controlled, multicenter, multinational ALSYMPCA trial enrolled patients from June 2008 through February 2011 [7]. Eligible patients had progressive metastatic symptomatic disease and regular use of analgesic medication or treatment with external beam radiation therapy (EBRT) for cancer-related bone pain within the previous 12 weeks. Patients were randomized 2:1 to receive six injections of radium-223 (50 kBq/kg IV) or a saline placebo infusion; a cycle was comprised of one injection every 4 weeks. The primary endpoint was overall survival (OS). Various secondary endpoints included time to first symptomatic skeletal events (SSE), time to alkaline phosphatase increase and time to PSA progression. A planned interim analysis was conducted, and on June 3, 2011, the Independent Data Monitoring Committee recommended stopping the trial early due to evidence of a significant treatment benefit. Median OS was 14.0 months for Ra-223 (95% CI (confidence interval): 12.1–15.8) versus 11.2 months for placebo (95% CI: 9.0–13.2) (*p* = 0.00185; HR (hazard ratio) = 0.695; 95% CI: 0.552–0.875).

An updated analysis included 528 events from 921 randomized patients prior to placebo patients crossing over to Ra-223 [7,261]. For this analysis, median OS was 14.9 months for Ra-223 arm (95% CI: 13.9–16.1) and 11.3 months for patients receiving placebo (95% CI: 10.4–12.8) (HR = 0.695; 95% CI: 0.581–0.832). OS favored Ra-223, with a 30% reduction in the risk of death compared with placebo. The OS benefit with Ra-223 was supported by a delay in time to first SSE compared to the placebo (15.6 versus 9.8 months; HR = 0.66; 95% CI, 0.52–0.83; *p* < 0.001). Improvement in survival was independent of both prior exposure to docetaxel [250] and the use of bisphosphonates [261]. Ra-223 significantly prolonged time to total ALP progression (HR = 0.167; 95% CI: 0.129–0.217; *p* < 0.00001); and time to PSA progression (HR = 0.643; 95% CI: 0.539–0.768; *p* < 0.00001) was associated with a beneficial effect on pain and quality of life (QOL) [262], and was associated with a highly favorable safety profile with a low rate of adverse hematological events. The most common (at least two percent) of the adverse reactions in patients receiving Ra-223 were nausea, diarrhea, vomiting, renal impairment and peripheral edema. 

Based on these data, the National Comprehensive Cancer Network (NCCN) supports the use of ^223^RaCl_2_ (Xofigo^®^) as a first-line therapy for patients with symptomatic metastatic CRPC and no visceral metastases with a Category 1 recommendation [227]. The recommended dose for ^223^RaCl_2_ is 50 kBq/kg (1.35 microcuries/kg) administered by slow intravenous injection, over 1 min, every 4 weeks, for a total of six cycles.

Since completion of the ALSYMPCA trial, Ra-223 has been studied in combination with other, newly-approved CRPC therapies, such as abiraterone, enzalutamide, docetaxel, cabazitaxel, sipuleucel-T, olaparib and pembrolizumab [263,264,265]. A phase 3, randomized, double-blind study of radium-223 or placebo, each in combination with abiraterone plus prednisone in chemotherapy-naive patients with asymptomatic or mildly symptomatic mCRPC with bone metastases (ERA 223; NCT02043678) [266], was recently prematurely unblinded. The independent data monitoring committee (IDMC) recommended unblinding the trial due to the observation of more fractures and deaths in the combination treatment arm. The European Medicines Agency (EMA)’s Pharmacovigilance Risk Assessment Committee (PRAC) has reviewed the preliminary data from the ongoing clinical study. In the study 34.7%, of patients treated with Xofigo, Zytiga and prednisone/prednisolone have died so far, compared with 28.2% of patients given placebo, Zytiga and prednisone/prednisolone. Fractures have also occurred more frequently with the Xofigo combination than the placebo combination (26% versus 8.1%). Unblinded data from the study are currently being analyzed to confirm the preliminary findings of the IDMC. Given these results from ERA 223 trial, the current recommendation is not to combine radium-223 with concomitant abiraterone acetate and prednisone (https://www.ema.europa.eu/news/prostate-cancer-medicine-xofigo-must-not-be-used-zytiga-prednisoneprednisolone). Hence, the administration of concomitant chemotherapy with Xofigo is not recommended due to an increased risk of adverse reactions [267]. 

Immune modulation has been examined in vitro in human prostate, breast and lung carcinoma cells following exposure to sub-lethal doses of ^223^Ra. Malamas and his colleagues showed that ^223^Ra significantly enhanced T cell-mediated lysis of each tumor type by CD8+ cytotoxic T lymphocytes (CTLs) specific for MUC-1, brachyury, and CEA tumor antigens. Therefore, ^223^Ra may be more effective in combination with immunotherapies [268].

In view of the increased long term risk of secondary malignancies, such as sarcomas and leukemias, that were seen in patients who were treated with high doses of another radium isotope, radium-224 (T_1/2_ = 3.7 days) (primarily used in Europe) for bone pain associated with ankylosing spondylitis, there is concern for development secondary malignancy with the use of Ra-223. So far there has been no evidence of secondary malignancies following Ra-223 therapy [7,269]. However, it is important to note that patients with metastatic CRPC have a much shorter overall survival than patients with ankylosing spondylitis. Care should be taken when expanding the use of Ra-223 to other patient groups with longer expected overall survival times, since the rate of secondary tumor development may increase. 

Ra-223 trials have been performed for patients with diseases other than CRPC. Ra-223 was found to be safe and well tolerated when studied in a phase II, open-label trial investigating the safety and short-term efficacy in patients with breast cancer who had bone-dominant disease [270], but the data are still limited. In contrast to prostate cancer, multiple myeloma bone lesions are predominantly lytic. It is not yet know if Ra-223 will have any role in this category of patients, but based on previous clinical trials using ^153^Sm-EDTMP plus chemotherapy, the combination showed efficacy in some patients with far-advanced myeloma [271], so theoretically, these results could be replicated with Ra-223, with potentially fewer side effects. In view of the limited but positive experience in ^153^Sm-EDTMP for osteosarcoma due to its osteoblastic nature [272], there is currently a phase I dose trial for the use of Ra-223 in patients with osteosarcoma at the M.D. Anderson Cancer Center (www.clinicaltrials.gov; NCT01833520). 

## 10. Conclusions

There has recently been significant interest and progression toward the development of targeted alpha-particle therapy (TAT) for the treatment of solid tumors. Many pre-clinical and clinical studies have demonstrated promising anti-tumor efficacy results (Table 2 and Table 3). The success of ^223^RaCl_2_ in the care of patients with bone metastases and the apparent efficacy of additional TAT agents in recent clinical studies, e.g., ^213^Bi–DOTATOC and ^225^Ac-PSMA, has brought attention to this area of radiopharmaceutical development and increased the potential for successful translation of TAT for treatment of solid tumors and metastases [65,233]. Three radioisotopes have seen the most use in preclinical and clinical studies, ^213^Bi, ^225^Ac and ^211^At. Each of these radionuclides has distinct advantages and disadvantages regarding availability, cost, decay chain attributes, tissue distribution and stability of attachment. Of these, ^225^Ac is readily available and has the distinct advantage of having a long, 10 d half-life, which allows for centralized production and distribution compared to the requirement of local production for the other radionuclides with shorter half-lives. Since TAT does not rely on the generation of free-radicals for the generation of DNA damage, as is the case for β-emission therapies, a major mechanism of resistance to radiation treatment can be bypassed; i.e., upregulation of superoxide dismutase. Some evidence of this was seen in a report stating that patients with tumors that have developed resistance to ^90^Y/^177^Lu–DOTATOC therapy were sensitive to subsequent treatment with ^213^Bi–DOTATOC [65]. Additionally, the use of α-emitters enables solid-tumor targeted therapy with decreased peripheral tissue damage and the potential for decreased development of resistance due to the ability to kill surrounding tumor cells that do not express the target marker in a heterogeneous tumor microenvironment. Small molecules, peptides and monoclonal antibodies have been used for tumor targeting (Table 2 and Table 3). However, due to the shorter clearance time (minutes to hours), small molecules and peptides may have an advantage over antibodies that can circulate for days in that they can accumulate in the target tumor and clear systemically prior to decay and deposition of energy, maximizing the beneficial effects of the ionizing radiation while minimizing the off-target exposure. This is especially true when using radionuclides with a long half-life; e.g., ^225^Ac. Peptide-targeted agents may have a greater risk of generating renal toxicity [66], but there is evidence that lead optimization via medicinal chemistry could potentially improve the renal toxicity of a given peptide agent. For example, a recent preclinical study of two different gastrin-releasing peptide receptor (GRP-R)-targeting agents using two different ^213^Bi-labeled GRP-R targeting peptides demonstrated differential renal toxicities [185]. TAT is currently emerging as a potentially new and effective approach toward the treatment of solid tumors, and the clinical translation of novel TAT radiopharmaceuticals appears to be on the near horizon. Since the abscopal effect has been observed in external beam therapy in animals, the ability to target internal radiation to tumors via TAT may have additional added value as a companion to the recently-successful immune checkpoint targeted therapies.

## Figures and Tables

**Table 1:** Physical characteristics of α-emitting radioisotopes investigated for clinical use.

Isotope	Half-Life	Max Energy	Emissions Per Decay
^225^Ac	10.1 d	5.83	4 α, 2β-
^211^At	7.2 h	5.87	1 α, 1 EC
^212^Bi	1.01 h	6.09	1 α, 1β-
^213^Bi	45.6 min	5.87	1 α, 2β-
^212^Pb	10.6 h	6.09	1 α, 2β-
^223^Ra	11.4 d	5.87	4 α, 2β-
^224^Ra	3.6 d	8.8	5 α, 2 β-
^149^Tb	4.1 h	3.96	1 α, 1 β+
^227^Th	18.7 d	6.04	5 α, 2β-

**Table 2:** Preclinical targeted alpha-particle therapy (TAT) studies.

Isotope	Study	Molecular Target	Targeting Moiety	Drug(s) & Route	Cancer Type & Animal Model	Key Results	Ref
^225^Ac	Efficacy, toxicity	PSMA, CD19	J591 & B4 mAbs	^225^Ac–DOTA–J591, ^225^Ac-B4, i.v.	Human LNCaP prostate s.c. xenografts & disseminated Daudi lymphoma in male nude mice.	Both effective without toxicity.	[35]
^225^Ac	Efficacy, toxicity	HER-2/neu	Trastuzumab	^225^Ac–DOTA–trastuzumab, i.p.	SKOV3 human ovarian cancer s.c. xenografts in female nude mice.	Effective with no toxicity.	[148]
^225^Ac	PK, RD, toxicity	CD33	HuM195 Ab	^225^Ac–DOTA–HuM195, i.v.	Cynomolgus monkey leukemia (does not express the human CD33 target).	12 d blood T_1/2_, dosimetry kinetics estimated, efficacy without renal toxicity.	[149]
^225^Ac	BD, efficacy, toxicity	Ganglioside GD2	3F8 Ab	^225^Ac–DOTA–3F8, i.v.	NMB-7 human neuroblastoma xenografts in nude mice (BD), meningeal carcinomatosis xenografts in nude rats (efficacy) & cynomolgus monkeys (toxicity).	Tumor specificity, increased survival, no toxicity.	[150]
^225^Ac, ^177^Lu	BD, efficacy, toxicity	Somatostatin receptors	DOTATOC peptide	^225^Ac and ^177^Lu–DOTATOC, i.v.	AR42J rat pancreatic exocrine s.c. xenografts in nude mice.	^225^Ac-TAT had greater efficacy relative to ^177^Lu-TBT with low toxicity.	[151]
^225^Ac, ^213^Bi, ^90^Y	BD, dosimetery, efficacy, toxicity	HER-2/neu	7.16.4 mAb	^225^Ac, ^213^Bi and ^90^Y-7.16.4, i.v.	neu-N transgenic mouse model with rat HER-2/*neu* expression and spontaneous lung metastases & NT2.5 mouse mammary fat pad xenografts with rat HER-2/*neu*.	^225^Ac-TAT had greater efficacy but with renal toxicity relative to ^213^Bi-TAT & ^90^Y-TBT.	[152]
^225^Ac, ^213^Bi	BD, efficacy, toxicity	nucleolin	F3 peptide	^225^Ac–DOTA–F3, ^213^Bi–DTPA–F3, i.p.	MDA-MB-435 human peritoneal carcinomatosis in SCID mice.	^225^Ac-TAT had greater efficacy relative to ^213^Bi-TAT with specific tumor uptake and minor renal toxicity.	[153,154]
^225^Ac	Vascular normalization & efficacy	Vascular endothelial (VE)-cadherin	E4G10 Ab	^225^Ac–DOTA–E4G10, i.v.	LS174T human colon s.c. xenografts in female nude mice.	Improved tumor vascular architecture & increased efficacy when combined with chemotherapy.	[107]
^225^Ac	Safety and efficacy	IL13RA2	Pep-1L peptide	[^225^Ac]Pep-1L, stereotactic intracranial injection	U8251 human glioblastoma orthotopic xenografts in male nude mice.	Efficacy with no significant toxicity.	[155]
^225^Ac	BBB and BTB permeabili-zation	Integrin α_v_β_3_	small-molecule antagonist	^225^Ac-labeled targeted liposomes (^225^Ac-TL), intracranial injection	U87 MG human glioblastoma orthotopic xenografts in male nude mice.	Enhanced blood-brain barrier (BBB) and bood-tumor barrier (BTB) permeability.	[156]
^225^Ac	BD, Efficacy	Thrombo-modulin	201b mAb	LnPO_4_ nanoparticles (NPs) doped with ^225^Ac-201b, i.v.	Syngeneic EMT6 mouse breast epithelial cell metastases in BALB/c mouse lung following i.v. injection of cells	Retention of ^225^Ac and daughters in lung tissue, metastasis burden reduced.	[157]
^225^Ac	Micro BD, RD	PD-L1	anti-PD-L1-BC Ab	^225^Ac–DOTA–anti-PD-L1-BC, i.v.	NT2.5 mouse mammary xenografts in female nude mice.	Uniform distribution in liver, non-uniform in kidney and tumor, liver RD was limiting.	[158]
^225^Ac	BD and toxicity	Bone metastasis	Zoledronic acid (ZOL)	^225^Ac–DOTAZOL, i.v.	Wistar rats.	High bone:blood ratio. Kidney toxicity.	[159]
^225^Ac	BD, RD and dose response	PSMA	PSMA ligands with albumin-binding moiety	^225^Ac-RPS-074, i.v.	LNCaP human prostate cancer s.c. xenografts in BALB/c mice.	Decreased clearance rate, single administration had complete response in 86% of tumors.	[160]
^225^Ac	PK, BD, specificity, RD, toxicity, efficacy	MC1R	MC1RL peptide	^225^Ac–DOTA–MC1RL, i.v.	PK (Sprague-Dawley rats), BALB/c mice (toxicity and BD) and MEL270 human uveal melanoma s.c. xenografts in SCID mice (BD and efficacy).	Renal and hepatobiliary excretion, rapid blood clearance, low toxicity, prolonged survival and decreased metastasis after single injection.	[67]
^225^Ac	Efficacy, toxicity	CA19.9	5B1 human mAb	^225^Ac-labeled tetrazine radioligand and a transcyclooctene5B1 for pretargeting, i.v.	Bilateral MIAPaCa-2 (CA19.9-negative) and BxPC3 (CA19.9-positive) pancreatic cancer s.c. xenografts, and BxPC3 orthotopic xenografts in nude mice.	Pretargeting has similar efficacy compared to conventional TAT with reduced hematotoxicity.	[144]
^211^At	BD, RD, specificity, efficacy, toxicity	Tenascin glycoprotein	81C6 mAb	^211^At-81C6, subarachnoid catheter or i.v.	Female athymic rat model of neoplastic meningitis by inoculation of human rhabdomyosarcoma cells via subarachnoid catheter.	Efficacy without significant toxicity. RD estimates.	[161,162]
^211^At	PK, BD, efficacy, toxicity	gp38	MOv18 mAb	^211^At- & ^131^I-MOv18, i.p. or i.v.	Peritoneal OVCAR-3 human ovarian xenografts in BALB/c ν/ν or nude mice following IP injection of cells.	^211^At-TAT had greater efficacy relative to ^131^I-TBT.	[163,164,165,166]
^211^At	Tumor neo-vasculature targeting	Fibronectin ED-B domain	Human scFv(L19)	^211^At-scFv(L19), i.v.	Murine F9 teratocarcinoma & rat FE8 sarcoma in female nude mice.	Retained at tumor blood vessels resulting in increased tumor to blood ratios.	[140]
^211^At	BD, tumor dosimetry, efficacy, toxicity	95-kDa glycoprotein	MX35 mAb	^211^At-MX35, i.p. or i.v.	OVCAR-3 human ovarian cancer micrometastases in nude mice.	Fractionated treatment increased efficacy without significant toxicity.	[23,167,168]
^211^A, ^90^Y	Efficacy	CD30	HeFi-1 mAb	^211^At-, ^90^Y HeFi-1, i.v.	Human anaplastic large cell lymphoma cells in SCID/NOD mice. Karpas 299 cell i.v. injection for leukemia & SUDHL-1 xenografts for lymphoma.	^211^At-HeFi-1 increased survival in the leukemia model & combination with unlabeled HeFI-1 further improved efficacy. ^90^Y-HeFi-1 TBT increased survival in the lymphoma model.	[169]
^211^At	BD, efficacy, toxicity	CD44v6	U36 chimeric mAb	^211^At-U36, i.v.	UT-SCC7 human head and neck squamous cell carcinoma s.c. xenografts in nude mice.	Reduced tumor growth with no significant toxicity. BD consistent with targeting.	[170]
^211^At	BD, efficacy, toxicity	HER2/neu	C6.5 diabody	^211^At-SAPS-C6.5, i.v.	HER2/neu-positive MDA-MB-361/DYT2 breast xenografts in nude mice.	Tumor growth delay with low renal toxicity.	[142]
^211^At	Efficacy	NIS-transduced tumor cells	Astatide (HAt) peptide	^211^At-astatide, i.p.	NIS transduced LNCaP human prostate (NP-1) and parental (P-1) s.c. xenografts in male nude mice.	Accumulation similar to iodine with efficacy against NP-1 tumors relative to control P-1 tumors.	[147]
^211^At, ^213^Bi	BD, myelo suppression, toxicity	CD45	30F11 Ab	^211^At-30F11-ADTM, ^213^Bi-30F11-CHX-A″, i.v.	Female BALB/c mice.	^211^At-TAT induced myeloablation in haematopoietic tissues with greater efficacy and less toxicity relative to the ^213^Bi conjugate.	[171]
^211^At	Efficacy	HER-2/neu	Trastuzumab	^211^At-trastuzumab, i.p.	SKOV3 human ovarian i.p. xenografts in nude mice.	Combination of trastuzumab and ^211^At-trastuzumab resulted in complete tumor eradication.	[172]
^211^At	Dosimetry, toxicity, efficacy	Lewis Y epitope	BR96, chimeric IgG1 mAb	^211^At-BR96, i.v.	BN7005-H_1_D_2_ rat syngeneic sub-peritoneal colon engraftments.	Resulted in undetectable tumors with tolerable toxicity.	[173]
^211^At	Efficacy	CD20	1F5 mAb	^211^At-1F5, i.v.	Human Ramos (Burkitt lymphoma) s.c. xenografts in nude mice and i.v. injection of Ramos cells in SCID mice for disseminated lymphoma.	Highly effective in minimal residual disease mouse model.	[77]
^211^At	BD, dosimetry	Sigma-2 receptor	MM3 ligand	^211^At-MM3, i.v.	EMT6 murine breast syngeneic tumor in female BALB/c mice.	Tumor specific targeting.	[146]
^211^At	Efficacy	Norepineph-erine transporter	Benzyl-guanidine	meta-[^211^At]-astatobenzyl-guanidine, i.v.	PC12 rat pheochromocytoma s.c. xenograft in nude mice.	Reduced tumor size without weight loss.	[174]
^211^At	BD, efficacy	MICA/B	anti MICA/B Ab	^211^At-anti MICA/B Ab, i.v.	HCT116 (p53^-/-^ & MICA/B positive) human colon cancer s.c. xenograft in nude mice.	Significant reduction in tumor growth, no weight loss, erythrocytopenia with recovery in 3-4 wks.	[175]
^213^Bi, ^90^Y	Toxicity and efficacy	CO17-1A	CO17-1A Fab’	^213^Bi-Fab’ and ^90^Y-Fab’, i.v.	GW-39 human colon cancer s.c. xenograft in nude mice.	TAT had greater efficacy and lower toxicity than TBT.	[176]
^212^Bi	Specificity, efficacy, toxicity	gp70	103A mAb	^212^Bi–CHX-A-DTPA–103A, i.v.	RLV induced erythroleukemia in BALB/c mice.	Clinical and histological remission of erythroleukemia and prolonged survival with low toxicity.	[177]
^212^Bi	BD, efficacy, toxicity	CD25	Anti-Tac, humanized mAb	^2l2^Bi–CHX-A-anti-Tac, i.v.	SP2 and SP2/Tac syngeneic murine lymphoma in nude mice.	Effective in treatment of bulky solid tumors.	[178]
^213^Bi	Stability, PK, toxicity	CD33	HuM195 mAb	^213^-Bi-CHX-A-DTPA–HuM195, i.p. or i.v.	Normal BALB/c mice without leukemia.	Favorable stability, PK and toxicity.	[179]
^213^Bi, ^90^Y	Pretargeting efficacy	CD25 (Tac)	Humanized anti-Tac mAb (HAT)	^213^Bi- & ^90^Y-DOTA–HAT; & HAT–streptavidin & ^213^Bi–DOTA–biotin or ^90^Y-DOTA–biotin, i.v.	Intraperitoneal MET-1 human adult T-cell leukemia in SCID/NOD mice.	Pre-targeted ^213^Bi TAT increased survival relative to ^213^Bi–DOTA–HAT, ^90^Y TBT & pre-targeted TBT.	[143]
^213^Bi, ^131^I	Efficacy	TAG-72	Humanized, domain-deleted CC49 mAb (HuCC49ΔCH2)	^213^Bi- or ^131^I-HuCC49ΔCH2, i.p.	TAG-72+ LS-174T & TAG-72 negative MIP human colon i.p. xenografts in nude mice.	^213^Bi-TAT had greater growth inhibition or regression relative to ^131^I-TBT.	[180]
^213^Bi	Efficacy, toxicity	d9-E-cad	d9-E-cad mAb	^213^Bi-d9-E-cad mAb, i.p.	HSC45-M2 human gastric i.p. xenografts with d9-E-cad mutation in female nude mice.	Double administration had greater efficacy relative to single administration, with no toxicity.	[181]
^213^Bi	BD, efficacy, toxicity	Somatostatin receptors	DOTATOC peptide	^213^Bi–DOTATOC, i.v.	CA20948 rat pancreatic adenocarcinoma tumors in Lewis rats.	Antitumor efficacy with low toxicity.	[64]
^213^Bi	Specificity, BD	CD87	P-P4D peptide	^213^Bi-P-P4D, i.p.	OV-MZ-6 human ovarian i.p. xenografts in female nude mice.	Specific tumor uptake, kidney uptake reduced by co-injection of gelofusine.	[182]
^213^Bi	Efficacy, toxicity	MUC1, uPAR and BLCA-38	C595 & BLCA-38 mAbs, & PAI2 protein	^213^Bi-C595, -BLCA-38 & -PAI2, i.p.	PC-3 human prostate orthotopic, intratibial and s.c. xenograft tumors in NOD SCID mice.	Multiple TAT can overcome heterogeneous antigen expression with efficacy against micrometastases.	[145]
^213^Bi	Efficacy, toxicity	EGFR	Matuzumab	^213^Bi-matuzumab, intravesical	EJ28 human orthotopic bladder xenografts in nude mice.	Increased survival without toxicity. Combination with mitomycin C increased efficacy with nephrotoxicity.	[183]
^213^Bi	Efficacy	TAG-72	Humanized CC49 mAb (HuCC49DCH2)	^213^Bi- HuCC49DCH2, i.p.	LS-174T human colon i.p. xenografts in female nude mice.	Combination trastuzumab and i.p. TAT increased efficacy and was well tolerated.	[184]
^213^Bi	Pretargeting efficacy	CD20	scFv-1F5-SA (streptavidin fusion protein)	1F5-SA & ^213^Bi–DOTA–biotin, i.v.	Ramos human lymphoma xenografts in nude mice.	Tumor regression and increased survival in mice with small tumors via pretargeting.	[141]
^213^Bi, ^177^Lu	BD, dosimetry, efficacy, toxicity	GRP	PESIN and AMBA peptides	^177^Lu–DOTA–PESIN, ^213^Bi–DOTA–PESIN, or ^213^Bi-AMBA, i.v.	PC-3 human prostate s.c. xenografts in female nude mice.	^213^Bi-TAT had greater efficacy compared to ^177^Lu-TBT. ^213^Bi–DOTA–PESIN had lower renal toxicity relative to ^213^Bi-AMBA.	[185]
^213^Bi, ^177^Lu	Efficacy	CD138	9E7.4 mAb	^213^Bi-9E7.4 and ^177^Lu-9E7.4, i.v.	5T33 murine multiple myeloma cell syngeneic i.v. injection into C57/BL6 mice.	^213^Bi-9E7.4 increased survival and cured 45%, ^177^Lu-9E7.4′ increased survival, no cures.	[186]
^213^Bi, ^177^Lu	Efficacy, toxicity	Mutant d9-E-cadherin	d9MAb	^213^Bi-d9Mab & ^177^Lu-d9Mab, i.p.	HSC45-M2 human gastric cancer cell i.p. injection in nude mice.	^213^Bi had comparable efficacy with lower toxicity.	[187]
^213^Bi	BD, efficacy, toxicity	CD138	Anti-mouse CD138 Ab	^213^Bi-CD138, i.v.	5T33 mouse multiple myeloma cell engraftment into syngeneic C57BL/KaLwRij mice.	Increased survival with only moderate and transient toxicity.	[188]
^213^Bi	Efficacy, toxicity	EGFR	Matuzumab	^213^Bi-matuzumab, intravesical.	EJ28 human orthotopic bladder xenografts in nude mice.	Increased survival with low toxicity.	[189]
^213^Bi	PK, efficacy, dosimetry, toxicity	SSTR2	DOTATATE peptide	^213^Bi–DOTATATE, i.v.	Neuroendocrine H69 human small cell lung carcinoma and CA20948 rat pancreatic s.c. xenografts in nude mice.	Effective in small and large tumors (both types), with dose limiting renal toxicity.	[66]
^212^Pb	Efficacy	HER-1	Cetuximab	^212^Pb-cetuximab, i.p.	ILS174T human colon i.p. xenografts in nude mice.	Extended survival and combined with gemcitabine & carboplatin increased efficacy.	[190]
^212^Pb	Efficacy	MC1R	DOTA-Re (Arg^11^)CCMSH peptide	^212^Pb[DOTA]–Re (Arg11)CCMSH, i.v.	B16/F1 murine melanoma syngeneic s.c. engraftments in C57BL/6 mice.	Tumor eradication at higher activities.	[191]
^212^Pb	Efficacy	HER-2 and CEA	Trastuzumab & 35A7	^212^Pb-trastuzumab & ^212^Pb-35A7, i.p.	A-431 HER-2 positive and CEA transfected vulvar squamous carcinoma cells i.p in nude mice.	Internalizing anti-HER2 labeled Ab had greater efficacy than non-internalizing anti-CEA labeled Ab.	[192]
^212^Pb	Efficacy, toxicity	HER-2/neu	Trastuzumab	^212^Pb-trastuzumab, i.p.	LS174T human colon & Shaw human pancreatic i.p. xenografts in nude mice.	Increased survival with low toxicity.	[193]
^212^Pb	Efficacy	HER-2/neu	Trastuzumab	^212^Pb-trastuzumab, i.p.	LS-174T human colon i.p. xenografts in nude mice.	Combination with gemcitabine increased survival.	[194]
^212^Pb	BD, efficacy	B7-H3	376.96 mAb	^212^Pb-376.96, i.p.	ES-2 or A2780cp20 human ovarian cancer cells i.p. into nude mice.	High peritoneal retention, tumor tissue accumulation & increased survival.	[195]
^212^Pb	BD, efficacy	B7-H3	376.96 mAb	^212^Pb-376.96, i.v.	Panc039 pancreatic cancer orthotopic xenografts in nude mice.	High tumor uptake & tumor growth inhibition.	[196]
^212^Pb	BD, efficacy	CSPG4	225.28 mAb	^212^Pb-225.28, i.v.	SUM159 & 2LMP human triple negative breast cancer (TNBC) orthotopic mammary fat pad xenografts in nude mice.	Dose-dependent growth inhibition.	[197]
^212^Pb	Administration route, toxicity, efficacy	EGFR	Panitumumab F(ab’)_2_ fragment	^212^Pb-panitumumab F(ab’)_2_, i.p. & i.v.	ILS-174T human colon i.p. xenografts in nude mice.	Increased survival with tolerated toxicity via i.p. or i.v. injection.	[198]
^212^Pb	Efficacy, combination therapy	MC1R	ee-cyclized α-MSH peptide	^212^Pb–DOTA–MC1L, BRAFi & HDACi	A2058 & MEWO human melanoma xenografts in nude mice.	Improved tumor response by combination therapy.	[69]
^227^Th	BD, efficacy, toxicity	CD20	Rituximab	^227^Th–DOTA–p-benzyl-rituximab, i.v.	BALB/c mice & Raji human B-cell lymphoma s.c. xenografts in nude mice.	Increased efficacy with managable toxicity.	[199]
^227^Th	BD, efficacy, toxicity	HER-2/neu	Trastuzumab	^227^Th–DOTA–trastuzumab, i.v.	SKBR-3 human breast cancer xenografts in nude mice.	Tumor growth inhibition with no toxicity.	[200]
^227^Th	BD, efficacy, toxicity	CD70	Anti-human CD70 mAb	CD70-TTC, i.v.	786-O human renal cancer s.c. xenografts in nude mice	Well tolerated with inhibition of tumor growth.	[201]
^224^Ra	Efficacy, toxicity	peritoneal metastases	Injection into peritoneum	^224^Ra-labeled calcium carbonate microparticles, i.p.	ES-2 and SKOV3 human ovarian cancer i.p. xenografts in nude mice.	Well tolerated with antitumor effect.	[202]

**Table 3:** Clinical TAT studies.

Isotope	Molecular Target	Targeting Moiety	Drug	Cancer Type	Trial/# of Patients	Administration Route	Key Results	Ref
^225^Ac	CD33	HuM195	^225^Ac–DOTA–HuM195	AML	Phase I/20, Ongoing multicentric phase I, II	Intravenous	Safe at doses ≤ 3 µCi/kg, anti-leukemic activity across all dose levels studied, no acute toxicities, myelosuppression	[235]
^225^Ac	PSMA	PSMA-617 ligand	^225^Ac–PSMA-617	Prostate cancer	NA/40	Intravenous	Remarkable anti-tumor response was observed in the patients. Xerostomia in salivary gland was the main side effect.	[5,6,60,233,236,237]
^225^Ac	Somatostatin receptors	DOTATOC peptide	^225^Ac–DOTATOC	Neuroendocrine tumors	NA/34	Not mentioned	Well-tolerated with promising treatment efficacy	[234]
^211^At	Tenascin-C	chimeric 816 antibody	^211^At-ch81C6	Glioblastoma	Phase I/18	surgically created resection cavity	Increased Median survival (54 weeks), No dose-limiting toxicity, No-grade 3 toxicity	[238]
^211^At	NaPi2b	MX35 F(ab′)2	^211^At-MX35 F(ab′)2	Ovarian carcinoma	Phase I/9	Intraperitoneal	No adverse effects, grade I toxicity, no bone marrow toxicity	[239]
^213^Bi	CD33	HuM195	^213^Bi–CHX-A-DTPA–HuM1 95	AML	Phase I/18	Intravenous	14 patients had reductions in marrow blasts	[240]
^213^Bi	CD33	HuM195	^213^Bi–CHX-A-DTPA–HuM1 95	AML	Phase I, II/31	Intravenous	dose-response relationship with remission at the highest doses	[241]
^213^Bi	CD20	Rituximab	^213^Bi-CHX-A”- Rituximab	Non-Hodgkin lymphoma	Phase I/9	Intravenous	Myelosuppression and no other toxic side, two patients responded	[242]
^213^Bi	Neurokinin type-1 receptor	Substance P	^213^Bi–DOTA–[Thi8, Met (O2) 11]–substance P	Glioblastoma	NA/2, 9, 20	Intrathecal	Well-tolerated, favorable response	[229,230,243]
^213^Bi	NG2 proteoglycan	9.2.27 antibody	^213^Bi–cDTPA–9.2.27	Melanoma	Phase I/38	Intralesional	TAT was safe up to 450 mCi and effective at 200 mCi	[244,245]
^213^Bi	Somatostatin receptors	DOTATOC peptide	^213^Bi–DOTATOC	Neuroendocrine tumors	NA/7	Intraarterial infusion	responses were observed in all patients	[65]
^213^Bi	EGFR	Cetuximab	^213^Bi–CHX-A-DTPA–anti-EGFR	carcinoma in situ (CIS) of the bladder	Pilot studies/9 and 12	Intravesical	TAT well tolerated and showed therapeutic efficacy	[231,232]
^212^Pb	HER2	Trastuzumab	^212^Pb–TCMC–trastuzumab	Ovarian Cancer	Phase I/3	Intraperitoneal	Dose escalation showed a little agent-related toxicity, consistent with the dosimetry data	[246,247]
^223^Ra	Hydroxy-apatite	NA	^223^Ra–chloride	Prostate cancer mets	Phase I-III/921	Intravenous	radium-223 improved overall survival	[7,8,82,248,249,250,251]
